# Personality and Meat Consumption Among Romantic Partners in Daily Life

**DOI:** 10.1111/jopy.12992

**Published:** 2024-11-11

**Authors:** Nicholas Poh‐Jie Tan, Maxim Trenkenschuh, Dana Ackermann, Leyla Anina Rosero Betancourt, Wiebke Bleidorn, Christopher J. Hopwood

**Affiliations:** ^1^ Department of Psychology The University of Zürich Zürich Switzerland

**Keywords:** daily diary, meat consumption, personality, romantic relationships

## Abstract

**Objective:**

Eating is often a social activity that can be influenced by others, particularly in close relationships when dietary preferences reflect underlying value differences. We sought to examine the personality traits of meat‐eating couples who differ in their preferences for meat.

**Method:**

We recruited Swiss romantic couples in which one partner typically consumed more meat than the other (*N* = 272, couples = 136). At baseline, participants completed survey measures of self‐ and informant‐rated personality traits at the domain (e.g., agreeableness) and aspect level (e.g., compassion) and meat consumption. Participants then completed 28 daily meal surveys about their meat consumption.

**Results:**

Among high‐meat eating partners, those higher in openness/intellect and compassion ate less meat. Additionally, higher intellect among low‐meat eating partners predicted lower meat consumption among high‐meat eating partners.

**Conclusions:**

These findings replicate evidence that personality plays an important role in meat‐eating and extend this evidence to meat consumption in a relational context.

## 1 Introduction

Eating is often a social activity in which people cook for one another and eat together. How, when, and what people eat can be influenced by others and perceived social norms (Higgs and Thomas 2016). This is particularly evident in romantic relationships where one individual eats more meat than the other, because the decision to eat or not eat meat can reflect strongly held values, attitudes, and motivations (Hopwood et al. 2021; Rozin, Markwith, and Stoess 1997). How individuals navigate such meal choices may be associated with personality. There is robust evidence that people who are higher in openness/intellect and agreeableness are more likely to endorse plant‐based diets (Reist et al. 2023). However, less focus has been paid to personality predictors of meat consumption at lower levels of the trait hierarchy. The goal of this study was to examine how these personality traits—at both the domain and aspect levels of the trait hierarchy—are related to meat consumption in couples who differ in their general preferences for meat.

### 1.1 The Social Context of Meat Consumption

Social influences on food consumption have been well documented. For instance, people have been found to eat more when eating in a group compared to alone, to model the consumption habits of others, and to moderate their consumption to manage their social impressions (for a review, see Higgs and Thomas 2016). When it comes to meat consumption, research highlights that the unavailability of plant‐based alternatives (Anderson and Milyavskaya 2022), gender role norms (Godin and Langlois 2021), and relational dynamics (Anderson and Milyavskaya 2022; Gregson and Piazza 2023) can act as barriers to meat reduction. Romantic relationships may provide a particularly important context for understanding the relation between personality and meat consumption. Previous research has shown that partners influence health‐related (Bartel et al. 2017; Monden, de Graaf, and Kraaykamp 2003; Stadler et al. 2023) and proenvironmental behaviors (Hung and Bayrak 2019; Seebauer, Fleiß, and Schweighart 2017). People in romantic relationships often share meals and must plan their food choices together. How they plan their food choices is particularly important when their food choices conflict—such as when one partner wants to eat meat but the other does not—as they can represent “fractures” in behavior that could promote longer term behavior change (O'Neill et al. 2019). For instance, Gregson and Piazza (2023) found that couples who perceived themselves as aligned in their dietary habits were less open to reducing their meat consumption compared to couples who did not. Thus, further understanding how couples navigate differences in food preferences is important for identifying the determinants of meat consumption.

The way this issue is navigated may be affected by the personalities of both individuals in a partnership. In addition to the associations of personality on choices to eat meat or not, some people may have the kinds of traits that make them resistant to influence by others and more likely to eat what they like, whether it is shared with partners or not. Others may have the kinds of traits that make them especially able to persuade their partners to eat what they, themselves, prefer. For example, Wortmann et al. (2023) found that individuals' food preferences impacted their own and their partner's diets. In this study, we examined how the personality traits of both people in a couple are associated with meat consumption for themselves and for their partners.

### 1.2 Personality and Meat Consumption

Traits predict a variety of important life outcomes (Roberts et al. 2007), including those related to health and well‐being (Strickhouser, Zell, and Krizan 2017), environmental concerns (Hopwood et al. 2024), and compassion for animals (Hopwood, Stahlmann, and Bleidorn 2023). Over the last decade, researchers have begun studying how personality traits, typically operationalized using the Big Five model, are related to meat consumption—usually defined in terms of vegetarian, vegan, or omnivore dietary identity.^1^ This work was summarized in a meta‐analysis by Reist et al. (2023), who found that higher openness/intellect or openness to experience and agreeableness distinguished vegetarians and vegans from omnivores, whereas only higher openness/intellect distinguished vegans from vegetarians. One potential reason we see those higher on openness/intellect being less likely to consume meat is that those higher on this trait tend to be more curious and reflective and to have more left‐leaning political attitudes (DeYoung 2014). Consequently, these individuals are more likely to be aware of and concerned about the environmental, animal welfare, and health issues associated with eating meat, and this may motivate them to eat less meat. For agreeableness, those higher on this trait tend to be more compassionate and empathetic (Kim and Han 2018), which research shows extends beyond human targets to animals (Paul 2000). Hence, these individuals may be more likely to eat less meat because of their sensitivity to the suffering of animals used to produce meat. Nevertheless, the research linking personality and meat consumption has four major limitations: These associations have not been examined in the context of close relationships; personality has been examined almost exclusively at the domain level; it has been assessed almost exclusively with self‐reports; and meat consumption has been conceptualized in terms of dietary identity.

#### 1.2.1 Personality Trait Domains and Aspects

Existing research on the associations between personality and meat consumption focuses almost exclusively on the Big Five domains of neuroticism, extraversion, openness/intellect, agreeableness, and conscientiousness. However, the Big Five model is hierarchical, such that each of these domains can be parsed into lower order traits. Doing so can help specify how personality traits are related to outcomes like meat consumption because domain‐level associations may mask nuance that is captured by these narrower traits.

For instance, Reist et al. (2023) found a somewhat weak association between agreeableness (i.e., the tendency to be considerate of others) and vegetarian diet (*d* = 0.17, 95% CI [0.09, 0.26]). However, Tan and colleagues (2021) found that vegetarians and vegans had higher levels of compassion (i.e., tendency for emotional affiliation with others) but not higher levels of politeness (i.e., tendency for reasoned affiliation with others; replicated in Hopwood et al. 2024). This finding suggests that only the compassionate aspects of agreeableness are related to meat consumption and highlights the value of distinguishing lower order traits. Likewise, Reist et al. (2023) found that neuroticism was not a correlate of vegetarian diet. However, neuroticism is sometimes found to be positively associated with vegetarianism (Forestell and Nezlek 2018) and other times negatively associated with vegetarianism (Pfeiler and Egloff 2020). This may suggest that some lower‐order features of neuroticism are related to meat consumption more than others.

Given that most existing research on individual differences in meat consumption has focused on the Big Five domains, in this study we aim to go one level of specificity further by examining the 10 aspects that can be used to distinguish different features of these domains. Neuroticism, or the tendency to experience negative emotions (e.g., anxiety, worry, and fear), can be parsed into the aspect traits volatility and withdrawal, which describe the external (e.g., anger) and internal (e.g., depression) expressions of negative affect, respectively (DeYoung 2015). Extraversion, or the tendency to experience positive affect and a sensitivity to rewarding stimuli (e.g., social interactions), can be split into enthusiasm and assertiveness (DeYoung, Quilty, and Peterson 2007). Openness/intellect describes a tendency to be imaginative, curious, and creative. This trait subsumes openness, or an interest in beauty, and intellect, or the pursuit of truth (DeYoung 2014). Finally, conscientiousness describes the tendency to be organized, dependable, and goal oriented with its two aspect traits, industriousness and orderliness, reflecting proactive and inhibitive approaches to goal pursuit (DeYoung, Quilty, and Peterson 2007).

#### 1.2.2 Self and Informant Perspectives on Personality

Most existing research on associations between personality and diet has used self‐report questionnaires to assess both traits and meat consumption. However, there are significant questions about how accurate people are in reporting about themselves within either domain (Dunning, Heath, and Suls 2004). With regard to meat consumption and human–animal relations more generally, some evidence suggests that people tend to downplay their meat consumption and its impacts (see Rothgerber 2019, 2020). With regard to personality, some traits are more amenable to self‐report than others, depending on factors such as how available and valid personality‐relevant cues are to others (Vazire 2010).

For these reasons, assessing personality via both self‐ and informant‐reports could not only shore up the shortcomings of both perspectives but also permit an examination of potentially interesting discrepancies (McAbee and Connelly 2016; Mõttus et al. 2020). The combination of self‐ and informant‐reports represents shared variance that many models of perception have defined as “accuracy” (e.g., Funder and West 1993). In contrast, self‐ and informant‐reports can help distinguish how people think about themselves (i.e., identity) as opposed to the impressions they make on others (i.e., reputation). Thus, examining self and other perspectives of personality, as we do in this study, can not only test the robustness of personality associations with meat consumption but also provide a more fine‐grained picture of these associations.

### 1.3 Conceptualizing Meat Consumption

In previous research on personality and plant‐based diet, meat consumption is commonly defined in terms of dietary identity (e.g., Forestell and Nezlek 2018; Pfeiler and Egloff 2018a). This approach has two distinct drawbacks. First, people are known to report being vegetarian even though they eat meat (Barr and Chapman 2002; de Boer, Schösler, and Aiking 2017; Gossard and York 2003; Krizmanic 1992; Rosenfeld and Tomiyama 2019; White, Seymour, and Frank 1999; Willetts 2013). This highly global indicator of meat consumption may thus not only capture what people actually eat but also how they want to see themselves, and they may be motivated by social desirability or other factors to claim to eat less meat than they do. Second, this categorical indicator may miss nuance in each of the dietary categories. For instance, meat reducers may describe themselves or be labeled by researchers as omnivores even though they eat meat substantially less often than other people in the same category. Similarly, there are those who would describe themselves as vegetarians but continue to occasionally eat meat (Rosenfeld and Tomiyama 2019).

To address these issues, we measured meat consumption as a continuous score that reflects how often people eat meat. Moreover, we assessed general meat consumption in a survey format and specific meat consumption during meals over the course of a 28‐day diary study. Cross‐sectional survey methods that ask participants to retrospect over a period of time (e.g., a week) and self‐report their general meat consumption have been commonly used when examining personality correlates of meat consumption (e.g., Forestell, Spaeth, and Kane 2012; Kessler et al. 2016; Mõttus et al. 2012, 2013; Pfeiler and Egloff 2018a, 2020). In contrast, daily diary or daily meal survey methods typically involve asking participants to self‐report their meat consumption once a day/meal for a number of days/meals. Cross‐sectional survey and daily meal survey methods have distinct advantages and disadvantages. For instance, compared to daily meal survey methods, survey methods are better able to capture how a person behaves in general across many contexts. However, such measures are susceptible to recall bias, which describes the errors in recall from forgetting an event or recalling an event that did not occur (Brusco and Watts 2015). In contrast, daily meal survey methods capture meat consumption as it occurs during actual meals, thereby mitigating recall bias. Moreover, daily meal survey methods are better able to capture important socio‐contextual factors—like having shared meals with a romantic partner—that are associated with meat consumption (Napa Scollon, Prieto, and Diener 2009). However, the inferences one can draw are limited to the contexts they sample. For example, if people tend to eat less meat during breakfast but only report what they eat during breakfast in a diary design, their meat consumption may be underestimated. Hence, combining both survey and daily meal survey methods could provide a more reliable measure of meat consumption that incorporates the strengths of both approaches. At the same time, exploring the associations between personality and both general and context‐specific meat consumption can help us better understand the impacts of factors such as identity, memory bias, and context.

### 1.4 The Present Study

In the present research, we aimed to identify the actor and partner effects of personality traits on meat consumption in romantic couples. We also sought to explore the impact of domain‐ and aspect‐level traits, self, and informant perspectives of personality, as well as general and context‐specific measures of meat consumption on these effects. As preregistered (https://osf.io/gx8ha), we hypothesized that higher levels of openness/intellect and agreeableness would predict lower meat consumption. We also preregistered several additional aims to explore partner effects of personality on meat consumption, as well as the effect of the other domain and aspect personality traits on meat consumption. We also tested the robustness of our effects to variations in our methodology, namely, if personality was measured using the self versus informant reports and if meat consumption was measured using the cross‐sectional baseline survey vs. daily meal survey.

## 2 Methods

### 2.1 Participants

The data for this study were collected as part of a broader project on romantic relationships, individual differences, and meat consumption (to see all variables measured, see codebook https://osf.io/wmk8q/). To be eligible for this study, participants had to be adults in a romantic partnership in which meals were commonly shared, and one partner had to eat more meat/animal products than the other. Critically, there was no eligibility criteria for partners to be cohabitating. However, couples were required to endorse eating meals together at least once per day. In total, 617 individuals from the Zürich region of Switzerland responded to online and physical advertisements to participate in a study titled “The Partner‐Diet Study,” in which they would be compensated 120 CHF for their full participation. Of these, 339 participants finished the baseline survey, and 282 completed the daily diaries. Of these 282, we excluded participants who failed both attention checks embedded in the baseline survey (*n* = 1), those who had not completed the personality measures (*n* = 1), those who were missing partner data (*n* = 2), and—because we are interested in dyads with differing levels of meat consumption—dyads who reported the same levels of meat consumption (*n* = 6). After these exclusions, the final sample size was *N* = 272 (136 dyads; female = 52%, *M*_*age*_ = 24.0, *SD*_*age*_ = 6.9). We ran a post hoc sensitivity analysis for distinguishable APIMs using the *APIMPowerR* shinyapp (Ackerman and Kenny 2016) and found that with our sample we had 80% power to detect an actor or partner effect of *β* = 0.16, assuming a *r* = 0.50 between traits. Demographic information can be found in the Tables S1 and S2 and study codebook (https://osf.io/wmk8q/).

### 2.2 Procedure and Measures

#### 2.2.1 Baseline Survey

Participants began the study by completing the baseline survey. This survey asked participants to report demographic information (e.g., age, gender, education, and income), their meat consumption, and their own and their partner's personalities. *General meat consumption* was measured by asking participants, “How often do you eat the following items … ” meat, fish, dairy, eggs, plant‐based meat alternatives, plant‐based dairy alternatives on a nine‐point scale ranging from 1 = never to 9 = with each meal? Because the present study is focused on meat consumption, we only used responses to “meat” and “fish” items, and a sum was computed across these items for each participant.

The German translation of the Big Five Aspect Scale (DeYoung, Quilty, and Peterson 2007) was used to measure personality. Each aspect‐level trait was measured with 10 items (e.g., “I love to reflect on things”), and all responses to these items were captured on a five‐point scale ranging from 1 = Do not agree at all to 5 = Fully agree. We adapted this same measure for the informant's reported personality (e.g., “My partner loves to reflect on things”). All personality domains and aspects were scored by computing a mean across relevant items. All domain and aspect traits for both the self and informant reported personality measures had internal consistency estimates (ω_t_) > 0.72 (see Table S3).

#### 2.2.2 Daily Meal Survey

Following the baseline survey, participants came to the laboratory, where they were given instructions on how to complete the daily meal survey measures. Participants were asked to complete two diaries in the laboratory and a further 26 once a day until they had completed 28 diaries in total. Each day, couples were required to agree on one shared meal for which they would both report. They then completed their rating for this meal separately (data were only used if both partners rated the same meal). To limit participant burden and ensure that all couples would report on shared meals, we had participants report one meal per day. Participants were sent reminders by the research team at 18:00 every day to complete ratings and further prompted if they had not completed the ratings by the end of the day. For response rates to daily diaries, please see the codebook (https://osf.io/wmk8q/). Participants were asked, “did you eat the following…” meat, fish, dairy, eggs, plant‐based meat alternatives, plant‐based dairy alternatives, vegetables, and fruits using a binary scale (yes/no). We computed each participant's *shared meat consumption* by calculating the proportion of meals for which meat or fish was consumed. We did not use a sum because not all dyads reported on the same number of meals.

### 2.3 Analysis Plan

As preregistered, we used Actor‐Partner Interdependence Models (APIM) to test our research questions. This approach allowed us to examine the effect of one's own personality trait level on their own meat consumption (i.e., actor effects) and on their partner's meat consumption (i.e., partner effects). Each model included one personality trait as the independent variable and meat consumption as the dependent variable. Because both general meat consumption (i.e., measured in the baseline survey) and shared meat consumption (i.e., measured in the meal‐level diary) were highly correlated (*r* = 0.71, 95% CI [0.65, 0.77], *p* < 0.001), we combined them by standardizing and summing them. Similarly, self‐ and informant‐reports of personality within dyads were highly correlated (see Table S4), so we combined them by computing mean scores across these reports for each participant. Both combined meat consumption and personality traits were used in the models to address our confirmatory hypothesis and exploratory aims to examine partner effects as well as domain and aspect traits. However, we also explored whether findings differed in survey‐ and meal‐level meat consumption data or self‐ and informant‐rated personality data.

Past research has found that vegetarians and vegans tend to be younger, more educated, and have higher incomes (Aston, Smith, and Powles 2013; de Boer, Schösler, and Aiking 2017; Stoll‐Kleemann and Schmidt 2017). Thus, we included these variables in each model as covariates. Gender is also associated with meat consumption (Stoll‐Kleemann and Schmidt 2017) and typically used as a distinguishing factor in APIMs applied to romantic couples. However, not all romantic couples in our sample were heterosexual, and gender was confounded with the fact that we recruited dyads in which each member had different levels of meat consumption. Women were much more likely to be the lower meat‐consuming member of heterosexual dyads (84%). Hence, we did not include gender as a covariate and used the differing levels of meat consumption between dyad members as the distinguishing factor. We did, however, conduct exploratory analysis in which we included gender as a covariate.

We empirically tested for distinguishability by first specifying an unconstrained distinguishable model that estimated separate actor and partner effects for each dyad member. We then specified an indistinguishable model in which we constrained the two actor and partner effects to be equal. As preregistered, we interpreted the model with a significantly greater fit of the data based on lower Bayesian Information Criterion (BIC) and Akaike Information Criterion (AIC; results of these model comparisons are summarized in Table S14). If these indicators conflicted, we interpreted the more parsimonious constrained indistinguishable model (results of the other models are reported in Table S16). We defined acceptable model fit as either a Goodness of Fit Index (GFI) ≥ 0.93 or an Standardized Root Mean Squared Residual (SRMR) ≤ 0.08 (Cho et al. 2020) and found that all models were deemed to have an acceptable fit (see Table S15 for model fit statistics). All analyses were conducted using R (R Core Team 2023), descriptive statistics were estimated using the *psych* package and functions (Revelle 2023), and APIMs were specified using Structural Equation Modeling with the *lavaan* package and functions (Rosseel 2012). We used an alpha of *p* < 0.05 and 95% confidence intervals to determine the statistical significance of our estimated parameters.

## 3 Results

Demographics can be found in Tables S1–S13, and descriptive statistics are summarized in Table 1. Examining the correlations within dyad revealed significant negative correlations between meat consumption and openness/intellect, but only for the partner who consumes more meat (*r*'s < − 0.17, *p's* < 0.05; see Table S6). Interestingly, the correlations for agreeableness were not significant for either partner (see Table S8).

**TABLE 1:** Descriptive statistics split by higher and lower meat consuming partner.

Variable	Partner	*M*	*SD*	Min	Max
Combined meat	Higher‐meat‐consumer	1.1	1.6	−2.1	4.8
	Lower‐meat‐consumer	−0.9	1.5	−2.5	2.9
General meat	Higher‐meat‐consumer	7.1	2.3	3	14
	Lower‐meat‐consumer	3.8	1.7	2	8
Meat (baseline survey)	Higher‐meat‐consumer	4.7	1.8	1	9
	Lower‐meat‐consumer	2.1	1.3	1	6
Fish (baseline survey)	Higher‐meat‐consumer	2.4	1.0	1	6
	Lower‐meat‐consumer	1.7	0.7	1	4
Shared meat	Higher‐meat‐consumer	0.4	0.2	0	1
	Lower‐meat‐consumer	0.2	0.3	0	1
Meat (daily meal survey)	Higher‐meat‐consumer	0.4	0.2	0	1
	Lower‐meat‐consumer	0.2	0.2	0	1
Fish (daily meal survey)	Higher‐meat‐consumer	0.1	0.1	0	0.4
	Lower‐meat‐consumer	0.1	0.1	0	1
Openness/intellect	Higher‐meat‐consumer	3.7	0.4	3.0	4.6
	Lower‐meat‐consumer	3.7	0.4	2.6	4.8
Openness	Higher‐meat‐consumer	3.6	0.5	2.0	4.9
	Lower‐meat‐consumer	3.5	0.6	2.0	4.7
Intellect	Higher‐meat‐consumer	3.9	0.5	2.6	4.9
	Lower‐meat‐consumer	3.8	0.5	2.6	5.0
Agreeableness	Higher‐meat‐consumer	3.9	0.4	2.8	4.6
	Lower‐meat‐consumer	4.0	0.4	2.9	5.0
Compassion	Higher‐meat‐consumer	4.1	0.4	2.9	5.0
	Lower‐meat‐consumer	4.2	0.4	2.8	5.0
Politeness	Higher‐meat‐consumer	3.8	0.4	2.6	4.6
	Lower‐meat‐consumer	3.8	0.4	2.4	4.6

*Note:* General meat is computed from the sum of meat and fish (baseline Survey); shared meat is computed from the proportion of meals when meat or fish was consumed (daily meal survey).

### 3.1 Openness/Intellect

**FIGURE 1:**
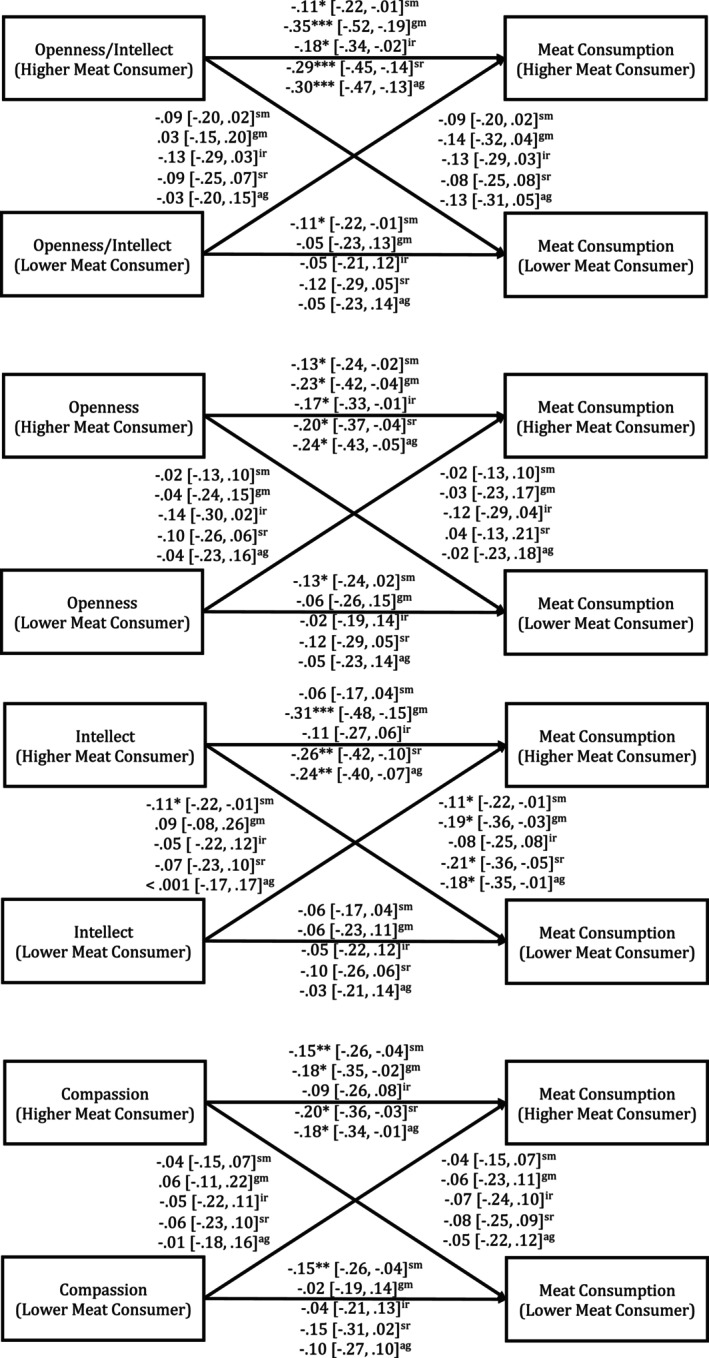
Summary of significant actor partner interdependence model parameters for personality predicting meat consumption. *Note:* ***p < 0.001, **p < 0.01, *p < 0.05; All effects are standardized; 95% confidence intervals are reported in square brackets; ^ag^ = aggregated personality and aggregated meat consumption; ^sr^ = self‐reported personality and aggregated meat consumption; ^ir^ = informant‐reported personality and aggregated meat consumption; ^gm^ = aggregated personality and general meat consumption; ^sm^ = aggregated personality and shared meat consumption. The models for shared meat consumption constrained actor and partner effects across higher and lower meat consumers; Effects on the left of each figure represent the partner effects for the dyad member who tends to consume more meat; Effects on the right of each figure represent the partner effects for the dyad member who tends to consume less meat. Covariates are included in the models but not reported in the figure for ease of presentation.

We first examined actor and partner effects of combined personality predicting combined meat consumption. As shown in Table 2 and Figure 1, we found the predicted significant actor effect for the dyad member who tended to consume more meat. This effect suggested that higher scores on openness/intellect predicted lower meat consumption. We found similar effects for the aspect traits openness and intellect. Specifically, we found significant actor effects for the dyad member who tended to consume more meat, which indicated that higher openness and intellect predicted lower meat consumption. We also found a significant partner effect that suggested that higher intellect for the dyad member who consumed less meat predicted lower meat consumption for the dyad member who consumed more meat. The remaining actor and partner effects for traits within the openness/intellect domain were not significant. See Figure S1 for a visual representation of these effects.

**TABLE 2:** Actor and partner effects of openness/intellect and agreeableness domain and aspect traits.

Model	Trait	Actor effect (higher meat consumer)				Actor effect (lower meat consumer)				Partner effect (higher meat consumer)				Partner effect (lower meat consumer)			
		*ß*	*p*	Lower CI	Higher CI	*ß*	*p*	Lower CI	Higher CI	*ß*	*p*	Lower CI	Higher CI	*ß*	*p*	Lower CI	Higher CI
Combined meat consumption	Openness/intellect	**−0.30**	**0.001**	**−0.47**	**−0.13**	−0.05	0.614	−0.23	0.14	−0.03	0.762	−0.20	0.15	−0.13	0.162	−0.31	0.05
	Openness	**−0.24**	**0.016**	**−0.43**	**−0.05**	−0.07	0.505	−0.27	0.13	−0.04	0.710	−0.23	0.16	−0.02	0.819	−0.23	0.18
	Intellect	**−0.24**	**0.006**	**−0.40**	**−0.07**	−0.03	0.712	−0.21	0.14	< 0.001	0.996	−0.17	0.17	**−0.18**	**0.042**	**−0.35**	**−0.01**
	Agreeableness	−0.13	0.146	−0.30	0.04	−0.10	0.266	−0.27	0.07	−0.02	0.852	−0.19	0.16	0.02	0.829	−0.15	0.19
	Politeness	−0.03	0.751	−0.21	0.15	−0.09	0.352	−0.27	0.10	−0.02	0.795	−0.21	0.16	0.10	0.272	−0.08	0.29
	Compassion	**−0.18**	**0.038**	**−0.34**	**−0.01**	−0.10	0.257	−0.27	0.07	−0.01	0.911	−0.18	0.16	−0.05	0.537	−0.22	0.12
Self‐report personality	Openness/intellect	**−0.29**	**< 0.001**	**−0.45**	**−0.14**	−0.12	0.161	−0.29	0.05	−0.09	0.286	−0.25	0.07	−0.08	0.323	−0.25	0.08
	Openness	**−0.20**	**0.013**	**−0.37**	**−0.04**	−0.12	0.159	−0.29	0.05	−0.10	0.238	−0.26	0.06	0.04	0.610	−0.13	0.21
	Intellect	**−0.26**	**0.001**	**−0.42**	**−0.10**	−0.10	0.238	−0.26	0.06	−0.07	0.420	−0.23	0.10	**−0.21**	**0.011**	**−0.36**	**−0.05**
	Agreeableness	−0.16	0.058	−0.32	0.01	−0.07	0.422	−0.24	0.10	0.02	0.820	−0.15	0.18	−0.01	0.952	−0.17	0.16
	Politeness	−0.09	0.284	−0.26	0.08	0.03	0.721	−0.14	0.20	0.09	0.297	−0.08	0.26	0.04	0.629	−0.13	0.21
	Compassion	**−0.20**	**0.020**	**−0.36**	**−0.03**	−0.15	0.088	−0.31	0.02	−0.06	0.459	−0.23	0.10	−0.08	0.367	−0.25	0.09
Informant‐report personality	Openness/intellect	**−0.18**	**0.028**	**−0.34**	**−0.02**	−0.05	0.563	−0.21	0.12	−0.13	0.113	−0.29	0.03	−0.13	0.123	−0.29	0.03
	Openness	**−0.17**	**0.034**	**−0.33**	**−0.01**	−0.02	0.788	−0.19	0.14	−0.14	0.082	−0.30	0.02	−0.12	0.150	−0.29	0.04
	Intellect	−0.11	0.203	−0.27	0.06	−0.05	0.566	−0.22	0.12	−0.05	0.549	−0.22	0.12	−0.08	0.319	−0.25	0.08
	Agreeableness	−0.05	0.580	−0.22	0.12	−0.08	0.329	−0.25	0.08	−0.10	0.250	−0.26	0.07	−0.01	0.945	−0.17	0.16
	Politeness	0.01	0.890	−0.16	0.18	−0.11	0.212	−0.27	0.06	−0.11	0.185	−0.28	0.05	0.06	0.445	−0.10	0.23
	Compassion	−0.09	0.315	−0.26	0.08	−0.04	0.635	−0.21	0.13	−0.05	0.515	−0.22	0.11	−0.07	0.422	−0.24	0.10
General meat consumption	Openness/intellect	**−0.35**	**< 0.001**	**−0.52**	**−0.19**	−0.05	0.571	−0.23	0.13	0.03	0.748	−0.15	0.20	−0.14	0.129	−0.32	0.04
	Openness	**−0.23**	**0.018**	**−0.42**	**−0.04**	−0.06	0.588	−0.26	0.15	−0.04	0.652	−0.24	0.15	−0.03	0.790	−0.23	0.17
	Intellect	**−0.31**	**< 0.001**	**−0.48**	**−0.15**	−0.06	0.491	−0.23	0.11	0.09	0.296	−0.08	0.26	**−0.19**	**0.024**	**−0.36**	**−0.03**
	Agreeableness	**−0.18**	**0.039**	**−0.35**	**−0.01**	−0.02	0.823	−0.19	0.15	0.07	0.396	−0.10	0.25	−0.02	0.833	−0.19	0.15
	Politeness	−0.13	0.171	−0.31	0.05	−0.02	0.850	−0.20	0.16	0.08	0.401	−0.11	0.26	0.04	0.704	−0.15	0.22
	Compassion	**−0.18**	**0.031**	**−0.35**	**−0.02**	−0.02	0.773	−0.19	0.14	0.06	0.510	−0.11	0.22	−0.06	0.482	−0.23	0.11
Shared meat consumption	Openness/intellect	**−0.11**	**0.039**	**−0.22**	**−0.01**					−0.09	0.115	−0.20	0.02				
	Openness	**−0.13**	**0.020**	**−0.24**	**−0.02**					−0.02	0.775	−0.13	0.10				
	Intellect	−0.06	0.247	−0.17	0.04					**−0.11**	**0.040**	**−0.22**	**−0.01**				
	Agreeableness	−0.11	0.057	−0.22	0.003					−0.01	0.917	−0.12	0.10				
	Politeness	−0.04	0.510	−0.15	0.07					0.03	0.612	−0.08	0.14				
	Compassion	**−0.15**	**0.009**	**−0.26**	**−0.04**					−0.04	0.490	−0.15	0.07				

*Note:* Bolded rows indicate statistically significant effects (i.e., *p* < 0.05); The APIM for shared meat consumption was indistinguishable and only one actor and partner effect for each trait is estimated; path coefficients are standardized.

We next examined whether the above findings differed when analyzing personality from the perspective of the self or the informant. Similar to the results outlined above, we found significant actor effects for the dyad member who tended to consume more meat, which suggested that higher self‐reported openness/intellect, openness, and intellect predicted lower meat consumption. We also found a significant partner effect that indicated that higher self‐reported intellect for the dyad member who tended to consume less meat predicted lower meat consumption for the dyad member who tended to consume more meat. For informant reports, we again found actor effects for the dyad member who tended to consume more meat and for openness/intellect and openness predicting meat consumption. The remaining actor and partner effects for self‐ and informant‐reported traits within the openness/intellect domain were not significant.

Finally, we also explored whether there were differences in effects across meat consumption in general and in the context of shared meals. For general meat consumption, we found actor effects for the dyad member that tended to consume more meat, which indicated that higher openness/intellect, openness, and intellect predicted lower meat consumption. We also found a significant partner effect, which suggested that higher intellect for the dyad member who consumed less meat predicted lower meat consumption for the dyad member who consumed more meat. For shared meat consumption, constrained indistinguishable models were fit, and we found significant actor effects, which suggested that higher openness/intellect and openness predicted lower meat consumption. The remaining actor and partner effects predicting general and context‐specific meat consumption were not significant.

### 3.2 Agreeableness

We first examined the actor and partner effects of self and informant reported agreeableness predicting meat consumption as measured in both the survey and the meal diaries (see Table 2). We did not find any significant actor or partner effects for agreeableness. However, when examining the agreeableness aspects, we found a significant actor effect for the dyad member who tended to consume more meat, which suggested that higher compassion predicted lower meat consumption (see Figure 1). The remaining actor and partner effects for traits within the agreeableness domain were not significant. See Figure S1 for a visual representation of these effects.

We next explored whether there were any actor or partner effects for self and informant reported traits within the agreeableness domain. For self‐report, we again found a significant actor effect for the dyad member who consumed more meat, which suggested that higher compassion predicted lower meat consumption. The remaining actor and partner effects for self‐reported agreeableness traits were not significant. Moreover, no actor or partner effects were found for informant‐reported traits within the agreeableness domain (see Table S15).

## 4 Discussion

The decision to eat or not eat meat can reflect strongly held values, attitudes, and motivations that must be navigated in relationships where one partner consumes more meat than the other (Hopwood et al. 2021; Rozin, Markwith, and Stoess 1997). The personalities of both partners are likely to shape how differences in meat‐eating tendencies are resolved during meal times. In this study, we aimed to identify the actor and partner effects of personality on meat consumption in romantic couples by examining (a) personality at different levels of abstraction, including Big Five domain traits and lower‐order aspect traits; (b) personality from the perspective of the self and relationship partners; and (c) meat consumption frequency in general and across 28 shared meals. There were three major findings.

First, consistent with previous findings, people who were more open to experience ate less meat. This finding was robust across reporters and measures of meat consumption. However, it was only present for members of the dyad who ate relatively more meat. Second, the compassion aspect of agreeableness was related to lower meat consumption, although the higher order domain and the politeness aspect were not. This finding was mostly consistent across measurement approaches but again only present for the members of the dyad who ate relatively more meat. Third, we found that people who are higher in intellect and lower in meat consumption than their partners have partners who eat less meat. However, this finding was only observed when low meat‐eating partners rated their own personality and for general and shared meat consumption.

### 4.1 Openness/Intellect

Previous research suggests that openness/intellect is the most robust predictor of lower meat consumption (Forestell, Spaeth, and Kane 2012; Forestell and Nezlek 2018; Kessler et al. 2016; Mõttus et al. 2013; Pfeiler and Egloff 2018a, 2018b, 2020; Reist et al. 2023; Tan et al. 2021), and this was generally replicated in this study. Moreover, this effect was consistently found when separately testing self‐ and informant‐reported openness/intellect, conceptualizing meat consumption in general and in the context of shared meals, and distinguishing openness and intellect aspects of the broad domain trait. Our design allowed us to tease apart two additional nuances to this finding.

First, it was only relevant for high meat eaters. A potential reason why we did not find any actor effects for dyad members who consumed less meat is because of constrained variability. Indeed, meat consumption for dyad members who consumed less meat had a slightly narrower range and smaller standard deviation than dyad members who consumed more meat (see Table S2). It is also possible that individuals who eat relatively more meat but are also high on traits within the openness/intellect domain consciously or unconsciously select partners who eat relatively less meat. It is also possible that individuals with higher openness/intellect are more likely to be persuaded by their partners or motivated to eat less meat to maintain their relationship.

Indeed, we also found a partner effect indicating that higher intellect for the dyad member who eats less meat predicts lower meat consumption for their partner who tends to eat more meat. Research shows that social support and control from romantic partners are effective ways to influence health‐related behaviors (Berli, Schwaninger, and Scholz 2021; Craddock et al. 2015), and evidence suggests those higher on openness/intellect tend to be perceived as more persuasive (Oreg and Sverdlik 2014). Moreover, openness/intellect is closely linked to creativity and creative problem solving (DeYoung 2014). It stands to reason that persuading people to eat less meat—a behavior that is difficult to change due to it being embedded in people's habits, rituals, and institutions (Bastian and Loughnan 2017)—requires a creative approach. Nevertheless, due to the novelty of this partner effect, replication in future research is required.

### 4.2 Agreeableness

We had hypothesized that higher agreeableness would predict lower meat consumption. However, effects were only observed for agreeableness with respect to general meat consumption as measured in the baseline survey. Although past research has found that vegetarians and vegans tend to be higher on agreeableness compared to omnivores (Keller and Siegrist 2015; Kessler et al. 2016; Pfeiler and Egloff 2020; Tan et al. 2021), Reist et al.'s (2023) meta‐analysis found only a weak effect for agreeableness—especially compared to the size of effect found for openness/intellect. Furthermore, all of these effects also occurred with general measures of diet assessed at the same time as traits, similar to the effect in our study.

Tan et al. (2021) suggested that it is important to distinguish different aspects of agreeableness. Consistent with this, we found a more robust effect for the compassion aspect of agreeableness than the politeness aspect. Given that compassion describes a tendency toward feelings of empathy and has been linked with compassion for animals specifically (DeYoung, Quilty, and Peterson 2007; Hopwood, Stahlmann, and Bleidorn 2023), it is possible that concerns over the suffering of farmed animals explain the association between compassion and lower meat consumption. Indeed, animal welfare concerns are one of the most cited motivations for adopting plant‐based diets (Hopwood et al. 2021).

### 4.3 Identity and Reputation

Some of our findings appeared to depend on the perspective of personality that we were examining. Specifically, we found that only self‐reported and not informant‐reported intellect and compassion were significant predictors of meat consumption. These findings suggest that how one thinks of themselves in terms of their intellect and compassion is more important for predicting meat consumption than what others think (McAbee and Connelly 2016). Indeed, such a notion dovetails with research highlighting how vegetarianism is more than just a diet and may be better conceptualized as an identity made up of various beliefs, attitudes, and motivations (Rosenfeld and Burrow 2017). Thus, our findings suggest that one's identity as intellectual and compassionate is more important than their reputation when it comes to meat reduction. This could be more closely examined by future research with the application of the Trait‐Reputation‐Identity Model (McAbee and Connelly 2016). This model explicitly separates the effects of self‐report (identity), informant‐report (reputation), and the shared variance between the two (trait) into latent factors to predict outcomes. Thus, this model would allow researchers to better test whether, for example, one's identity when it comes to intellect and compassion are more relevant than their reputation or trait when predicting meat consumption.

Another potential explanation for why we found effects for self‐reported but not informant‐reported intellect and compassion is that self‐reported personality and general meat consumption share method variance. Therefore, the effect could be due to similarities between the methods used to measure personality and meat consumption. For instance, the differences in self‐ and informant‐reported intellect and compassion could be explained by social desirability that is correlated between self‐reports of personality and general meat consumption—which makes up the combined meat consumption variable.

### 4.4 General and Shared Meat Consumption

Some of our findings also seemed to differ depending on whether we examined general or shared meat consumption. Specifically, we found a strong actor effect of intellect predicting general meat consumption, but not when examining shared meat consumption. One possible explanation is that sharing meals with a romantic partner does not afford the expression of intellect (Reis 2008). In other words, the ability to understand complicated abstract ideas might be important for predicting one's own meat consumption in general (e.g., the impact of meat consumption on climate change; DeYoung 2014). However, when it comes to the coordination of shared meals with one's romantic partner, this ability might matter less. Differences in findings between general and shared meat consumption could also be due to method effects, in that personality and general meat consumption were measured at the same time, whereas daily meals were rated afterward.

### 4.5 Implications

The present research has both theoretical and practical implications. An important goal of personality psychology is the comprehensive and detailed description of associations between personality and behavioral outcomes (Mõttus et al. 2020). To that end, we contributed to the description of the association between personality and meat consumption by going beyond the “big few” domain traits and examining the role of aspect‐level traits. This highlights the value of examining lower‐order traits and provides information for researchers and practitioners about the kinds of traits that best predict meat consumption. Moreover, we explored the generalizability of our findings across different contexts and methodologies. For example, we found that the effect between intellect and meat consumption was only found for general meat consumption and self‐reported intellect. Thus, the present research contributes a deeper understanding of how individual differences in personality are related to a common, psychologically interesting, and consequential behavior.

The present research also has implications for the understanding of meat consumption in the context of romantic relationships. The planning and sharing of meals are a common occurrence for romantic couples and can be a source of conflict when one partner wants to eat meat and the other does not. Given that meat‐eating for some is a value‐laden behavior (Rozin, Markwith, and Stoess 1997), navigating this conflict may be a particularly salient challenge for couples that could require compromise. In extreme cases, this could mean a vegetarian must cook meat or an omnivore must consume tofu. Indeed, how couples manage this challenge might depend on the personalities of both partners. In the present research, we found that the openness/intellect and compassion of the partner who tended to consume more meat significantly predicted reduced meat consumption. Thus, it appears either that these traits are related to how partners select one another or that traits predict which individuals tend to compromise their meat consumption to align better with their partners.

Our research also has more practical implications for policymakers as well as environmental and animal rights activists seeking to reduce meat consumption. Specifically, the findings of the present research could help inform more effective, personalized, and targeted interventions by identifying the traits that are associated with meat consumption. For example, Matz and Colleagues (2017) found that persuasive appeals designed to target those high on openness by emphasizing creativity and intellectual stimulation were more effective at influencing behavior. Given our findings concerning traits within the openness/intellect domain and compassion, a similar intervention promoting curiosity to learn about the impacts of meat consumption on animal welfare could be effective at reducing meat consumption (Ammann et al. 2023).

### 4.6 Limitations and Future Research

Several study limitations and future directions for research should be noted. First, our focus on couples who consumed differing amounts of meat limited the generalizability of our findings. This sampling approach limits the generalizability of our findings to other kinds of romantic couples (e.g., those who consume similar amounts of meat) and dyads (e.g., friends and family). However, when we constrain the effects for higher and lower meat consumers, we find a largely similar pattern of results (see Table S16). This suggests that the effects found for higher meat consumers may extend to couples who also eat similar levels of meat. Another consequence of focusing on couples with differing levels of meat consumption is that most of the dyads in our sample were heterosexual, with higher and lower meat consumers tending to be men and women, respectively. While this configuration is representative of most couples around the world, our findings remain largely limited to heterosexual couples in which men consume more meat than women. It is, however, somewhat reassuring that our exploratory APIMs, which include gender as a covariate, yield a similar pattern of results (see Table S17). Future research could seek to extend the effects found in the present study to same‐sex couples, couples of varying relationship length, or couples who consume similar amounts of meat but have agreed to try to reduce their consumption together.

While our selective criteria of recruiting dyads who consumed differing levels of meat allowed us to examine personality influences on meat consumption in a novel social context, it also introduced the potential for selection bias. Specifically, distinguishing partners based on their level of meat consumption could lead to spurious associations between personality and meat consumption (Elwert and Winship 2014). Reassuringly, many of the effects found in the present study appear to replicate previous research findings. Nevertheless, future research could mitigate this bias by more randomly sampling romantic couples based on their meat consumption.

Another limitation of the present research was the focus on a culturally homogenous sample. Our sample consisted of German‐speaking adults from the Zürich region of Switzerland, most of whom were heterosexual. While it is reassuring that some of our findings replicate previous research that has focused on more diverse samples (e.g., older Americans; Tan et al. 2021), it remains unclear whether our findings would generalize to other populations around the world.

Another limitation has to do with opportunities for the expression of specific personality traits. Personality can influence diet in various ways, and their observability might depend on culture and context. For instance, cultures and contexts with strong norms around eating meat might afford the expression of neuroticism, which describes a sensitivity to threat and punishment that may arise from norm‐violations (DeYoung, Quilty, and Peterson 2007). Thus, to better understand how personality relates to meat consumption, it will be important for future research to study other cultures and contexts in which meat is consumed.

Our research was also limited because of our focus on domain‐ and aspect‐level traits. It is possible that lower order trait facets or nuances (e.g., individual items) (Mõttus et al. 2017; Soto and John 2017) would provide an even greater level of specificity about how personality is related to meat consumption. For instance, Tan et al. (2021) found that vegetarians and vegans were significantly higher on the trust facet of agreeableness compared to omnivores. As noted in the introduction, ascertaining the level of the trait hierarchy that best explains the relationship between personality and meat consumption is important for fully understanding any given phenomenon (Mõttus et al. 2020). Thus, future research could benefit from an investigation of how lower‐level traits are related to meat consumption.

As discussed in the introduction, there are advantages and disadvantages to the use of cross‐sectional survey and daily meal survey methods that we sought to balance by combining the two. However, a prevailing disadvantage is that both measures were still self‐reported. Self‐report measures can be susceptible to social desirability and demand characteristics. Indeed, previous research has shown that feelings of discomfort caused by the moral implications of meat‐eating can lead people to misreport their actual meat consumption (Rothgerber 2020). Thus, future research should employ measures of actual meat consumption.

### 4.7 Conclusion

In summary, we sought to identify the associations between the personality traits openness/intellect and agreeableness and meat consumption among romantic partners. We did so by exploring actor and partner effects, domain‐ and aspect‐level traits, personality from the perspective of the self and one's partner, and general and shared meat consumption. We found that higher openness/intellect, openness, intellect, and compassion were associated with lower meat consumption, but only for the partner who consumed more meat. We also found that higher intellect for the partner who tended to eat less meat predicted lower meat consumption for the partner who tended to consume more meat. Finally, our findings for intellect and compassion appeared to differ based on the perspective of personality and whether we focused on general or shared meat consumption—the latter only applied to intellect. These findings replicate previous studies that indicated the importance of openness/intellect and agreeableness for a vegetarian or vegan diet, extend these findings to more direct and continuous measures of meat consumption, and add nuance regarding the roles of self‐ and informant‐perspectives on personality, meat consumption in general as opposed to within specific meals, and the specific relevance of these traits for people who tend to eat more as opposed to less meat within romantic relationships.

## Author Contributions

The research idea was conceived of by N.P.‐J.T. and C.J.H. Data collection was completed by M.T., D.A., and L.A.R.B. N.P.‐J.T. analysed the data and wrote the initial version of the manuscript. All authors revised the manuscript and approved the final version.

## Ethics Statement

The study protocol was approved by the Ethics Committee of the Department of Psychology at the University of Zürich, Switzerland (approval no. 22.2.9).

## Conflicts of Interest

The authors declare no conflicts of interest.

## Supporting information


Data S1.

## Data Availability

All materials, data, and analysis scripts used in the present research can be accessed at https://osf.io/gx8ha.

## Endnotes

^1^
A vegetarian is a person who identifies as someone who does not eat animal meat but does eat other animal products like dairy and eggs. A vegan is a person who identifies as someone who does not eat any animal product at all. Finally, an omnivore is a person who eats animals.
