# Supplementary material for: Personality and Meat Consumption Among Romantic Partners in Daily Life
Source: J Pers. 2024 Nov 11;93(5):1067–80. doi: 10.1111/jopy.12992 (PMC12421718; doi:10.1111/jopy.12992)
Supplement: Supplementary file 1 — Data S1. [file JOPY-93-1067-s001.docx]

# Supplementary Materials

## Table S1

*Categorical Demographic Split by Partner’s Level of Meat Consumption.*

| Variable |  | Higher-Meat-Consumer | Lower-Meat-Consumer |
| --- | --- | --- | --- |
| Education | Bachelor | 27 | 26 |
|  | Diploma Higher Technical College | 2 | 0 |
|  | Federal Certificate/Federal Diploma | 2 | 1 |
|  | Federal Certificate of Competence | 13 | 2 |
|  | High School Diploma | 63 | 82 |
|  | Master | 12 | 10 |
|  | Other | 6 | 5 |
|  | PhD | 1 | 3 |
|  | Technical Secondary School/Commercial Secondary School/IT Secondary School | 3 | 0 |
|  | Vocational Baccalaureate | 7 | 7 |
| Gender | Female | 28 | 114 |
|  | Male | 108 | 21 |

*Note.* These demographics are based on the final sample size *N* = 272; One participant did not report their gender.

## Table S2

*Continuous Demographics Split by Partner’s Level of Meat Consumption.*

| Variable | Partner | *N* | *M* | *SD* | Min | Max | Skew | Kurtosis |
| --- | --- | --- | --- | --- | --- | --- | --- | --- |
| General Meat | Higher-Meat-Consumer | 136 | 7.1 | 2.3 | 3 | 14 | .55 | -.31 |
|  | Lower-Meat-Consumer | 136 | 3.8 | 1.7 | 2 | 8 | .57 | -.85 |
| Meat (Baseline Survey) | Higher-Meat-Consumer | 136 | 4.7 | 1.8 | 1 | 9 | .35 | -.57 |
|  | Lower-Meat-Consumer | 136 | 2.1 | 1.3 | 1 | 6 | .85 | -.36 |
| Fish (Baseline Survey) | Higher-Meat-Consumer | 136 | 2.4 | 1.0 | 1 | 6 | 1.17 | 2.05 |
|  | Lower-Meat-Consumer | 136 | 1.7 | .7 | 1 | 4 | .97 | .73 |
| Shared Meat | Higher-Meat-Consumer | 136 | .4 | .2 | 0 | 1 | .06 | -.96 |
|  | Lower-Meat-Consumer | 136 | .2 | .3 | 0 | 1 | .84 | -.53 |
| Meat (Daily Diary) | Higher-Meat-Consumer | 136 | .4 | .2 | 0 | 1 | .29 | -.75 |
|  | Lower-Meat-Consumer | 136 | .2 | .2 | 0 | 1 | 1.20 | .56 |
| Fish (Daily Diary) | Higher-Meat-Consumer | 136 | .1 | .1 | 0 | .4 | 1.63 | 2.94 |
|  | Lower-Meat-Consumer | 136 | .1 | .1 | 0 | 1 | 4.47 | 30.35 |
| Age | Higher-Meat-Consumer | 136 | 24.7 | 7.6 | 18 | 67 | 3.32 | 13.01 |
|  | Lower-Meat-Consumer | 136 | 23.3 | 6.0 | 18 | 57 | 3.84 | 18.11 |
| Income | Higher-Meat-Consumer | 128 | 31657 | 53671 | 0 | 500000 | 5.48 | 43.01 |
|  | Lower-Meat-Consumer | 120 | 15236 | 20818 | 0 | 95000 | 1.97 | 3.39 |

*Note.* Income is in CHF.

## Table S3

*McDonalds Omega (ω_t_) and Cronbach’s Alpha (α) for Self-Reported, Informant-Reported, Aggregated Personality Traits.*

| Trait | Measure | *ω_t_* | *α* |
| --- | --- | --- | --- |
| Openness/Intellect | Self-reported | .80 | .73 |
|  | Informant-reported | .88 | .84 |
|  | Aggregated | .84 | .80 |
| Openness | Self-reported | .84 | .79 |
|  | Informant-reported | .87 | .80 |
|  | Aggregated | .93 | .90 |
| Intellect | Self-reported | .82 | .78 |
|  | Informant-reported | .86 | .83 |
|  | Aggregated | .85 | .81 |
| Agreeableness | Self-reported | .85 | .82 |
|  | Informant-reported | .87 | .84 |
|  | Aggregated | .87 | .84 |
| Compassion | Self-reported | .89 | .86 |
|  | Informant-reported | .86 | .83 |
|  | Aggregated | .82 | .71 |
| Politeness | Self-reported | .69 | .63 |
|  | Informant-reported | .77 | .72 |
|  | Aggregated | .79 | .75 |
| Conscientiousness | Self-reported | .90 | .87 |
|  | Informant-reported | .91 | .89 |
|  | Aggregated | .87 | .82 |
| Industriousness | Self-reported | .87 | .85 |
|  | Informant-reported | .87 | .84 |
|  | Aggregated | .87 | .82 |
| Orderliness | Self-reported | .85 | .80 |
|  | Informant-reported | .89 | .85 |
|  | Aggregated | .83 | .75 |
| Neuroticism | Self-reported | .93 | .91 |
|  | Informant-reported | .94 | .93 |
|  | Aggregated | .92 | .89 |
| Withdrawal | Self-reported | .89 | .86 |
|  | Informant-reported | .90 | .88 |
|  | Aggregated | .87 | .80 |
| Volatility | Self-reported | .89 | .87 |
|  | Informant-reported | .92 | .90 |
|  | Aggregated | .89 | .83 |
| Extraversion | Self-reported | .89 | .86 |
|  | Informant-reported | .89 | .87 |
|  | Aggregated | .85 | .80 |
| Enthusiasm | Self-reported | .87 | .82 |
|  | Informant-reported | .88 | .84 |
|  | Aggregated | .82 | .74 |
| Assertiveness | Self-reported | .85 | .81 |
|  | Informant-reported | .84 | .81 |
|  | Aggregated | .77 | .67 |

## Table S4

*Pearson Correlations between Self and Informant Reported Personality Traits for Higher and Lower Meat Consumers.*

|  | Higher Meat Consumer | | |  | Lower Meat Consumer | | |
| --- | --- | --- | --- | --- | --- | --- | --- |
| Trait | *r* | Upper CI | Lower CI |  | *r* | Upper CI | Lower CI |
| Openness/Intellect | .55 | .42 | .66 |  | .44 | .29 | .57 |
| Openness | .65 | .54 | .74 |  | .58 | .45 | .68 |
| Intellect | .39 | .23 | .52 |  | .40 | .24 | .53 |
| Agreeableness | .36 | .20 | .50 |  | .33 | .17 | .47 |
| Compassion | .31 | .15 | .45 |  | .45 | .30 | .57 |
| Politeness | .52 | .38 | .63 |  | .32 | .16 | .46 |
| Neuroticism | .47 | .33 | .59 |  | .64 | .53 | .73 |
| Withdrawal | .45 | .30 | .57 |  | .65 | .54 | .74 |
| Volatility | .46 | .32 | .59 |  | .55 | .42 | .66 |
| Extraversion | .44 | .29 | .57 |  | .44 | .29 | .56 |
| Enthusiasm | .42 | .27 | .55 |  | .36 | .21 | .50 |
| Assertiveness | .47 | .33 | .59 |  | .49 | .35 | .61 |
| Conscientiousness | .62 | .50 | .71 |  | .57 | .44 | .67 |
| Orderliness | .54 | .40 | .65 |  | .51 | .38 | .63 |
| Industriousness | .55 | .42 | .65 |  | .52 | .39 | .64 |

*Note.* 95% Confidence intervals are reported; All correlations are *p* < .001.

## Table S5

*Pairwise Pearson Correlations and Descriptive Statistics for Meat Consumption and Traits Within the Openness/Intellect Domain Split by Measurement Method.*

|  | 1. |  | 2. |  | 3. |  | 4. |  | 5. |  | 6. |  | 7. |  | 8. |  | 9. |  | 10. |  | 11. |  | 12. |
| --- | --- | --- | --- | --- | --- | --- | --- | --- | --- | --- | --- | --- | --- | --- | --- | --- | --- | --- | --- | --- | --- | --- | --- |
| 1. Combined Meat |  |  |  |  |  |  |  |  |  |  |  |  |  |  |  |  |  |  |  |  |  |  |  |
| 2. General Meat | .93 | [.91, .94] |  |  |  |  |  |  |  |  |  |  |  |  |  |  |  |  |  |  |  |  |  |
| 3. Shared Meat | .93 | [.91, .94] | .72 | [.66, .77] |  |  |  |  |  |  |  |  |  |  |  |  |  |  |  |  |  |  |  |
| 4. Openness/Intellect (aggregated) | -.15 | [-.26, -.03] | -.16 | [-.27, -.04] | -.12 | [-.24, -.001] |  |  |  |  |  |  |  |  |  |  |  |  |  |  |  |  |  |
| 5. Openness/Intellect (self-reported) | -.22 | [-.33, -.10] | -.21 | [-.33, -.10] | -.19 | [-.30, -.07] | .77 | [.72, .82] |  |  |  |  |  |  |  |  |  |  |  |  |  |  |  |
| 6. Openness/Intellect (partner-reported) | -.03 | [-.15, .09] | -.04 | [-.16, .08] | -.01 | [-.13, .11] | .81 | [.77, .85] | .26 | [.15, .37] |  |  |  |  |  |  |  |  |  |  |  |  |  |
| 7. Openness (aggregated) | -.11 | [-.23, .01] | -.10 | [-.22, .02] | -.11 | [-.22, .01] | .84 | [.80, .87] | .67 | [.60, .73] | .66 | [.59, .73] |  |  |  |  |  |  |  |  |  |  |  |
| 8. Openness (self-reported) | -.20 | [-.31, -.08] | -.19 | [-.31, -.08] | -.18 | [-.29, -.06] | .64 | [.56, .70] | .83 | [.79, .87] | .20 | [.09, .32] | .78 | [.73, .82] |  |  |  |  |  |  |  |  |  |
| 9. Openness (partner-reported) | .02 | [-.10, .14] | .04 | [-.08, .15] | .01 | [-.11, .13] | .68 | [.61, .74] | .23 | [.11, .34] | .83 | [.79, .86] | .79 | [.74, .83] | .23 | [.11, .34] |  |  |  |  |  |  |  |
| 10. Intellect (aggregated) | -.13 | [-.25, -.01] | -.16 | [-.27, -.04] | -.08 | [-.20, .04] | .76 | [.71, .81] | .56 | [.48, .64] | .65 | [.57, .71] | .29 | [.18, .40] | .19 | [.07, .30] | .27 | [.15, .37] |  |  |  |  |  |
| 11. Intellect (self-reported) | -.13 | [-.25, -.01] | -.14 | [-.25, -.02] | -.11 | [-.22, .01] | .57 | [.48, .64] | .72 | [.66, .77] | .20 | [.09, .31] | .21 | [.09, .32] | .22 | [.10, .33] | .12 | [-.003, .23] | .75 | [.70, .80] |  |  |  |
| 12. Intellect (partner-reported) | -.08 | [-.19, .04] | -.11 | [-.23, .004] | -.03 | [-.15, .09] | .62 | [.54, .69] | .19 | [.07, .30] | .77 | [.72, .82] | .24 | [.13, .35] | .09 | [-.03, .21] | .29 | [.18, .39] | .81 | [.76, .84] | .22 | [.10, .33] |  |
| Mean | .1 |  | 5.4 |  | .3 |  | 3.7 |  | 3.7 |  | 3.7 |  | 3.5 |  | 3.6 |  | 3.5 |  | 3.9 |  | 3.8 |  | 3.9 |
| SD | 1.8 |  | 2.6 |  | .3 |  | .4 |  | .5 |  | .5 |  | .5 |  | .7 |  | .7 |  | .5 |  | .6 |  | .6 |
| Min | -2.5 |  | 2.0 |  | 0 |  | 2.6 |  | 2.5 |  | 2.4 |  | 2.0 |  | 1.8 |  | 1.6 |  | 2.6 |  | 2.1 |  | 1.6 |
| Max | 4.8 |  | 14.0 |  | 1.0 |  | 4.8 |  | 4.8 |  | 4.8 |  | 4.9 |  | 5.0 |  | 4.9 |  | 5.0 |  | 5.0 |  | 5.0 |

*Note.* 95% Confidence Intervals are reported in square brackets; Combined meat consumption is comprised of general and shared meat consumption; General meat consumption is measured using the baseline survey; Shared meat consumption is measured using daily diaries.

## Table S6

*Pairwise Pearson Correlations and Descriptive Statistics for Meat Consumption and Traits Within the Openness/Intellect Domain Split by Level of Meat Consumption.*

|  | 1. |  | 2. |  | 3. |  | 4. |  | 5. |  | 6. |  | 7. |  | 8. |
| --- | --- | --- | --- | --- | --- | --- | --- | --- | --- | --- | --- | --- | --- | --- | --- |
| 1. Meat Consumption (Higher Meat Consumer) |  |  |  |  |  |  |  |  |  |  |  |  |  |  |  |
| 2. Meat Consumption (Lower Meat Consumer) | .61 | [.49, .71] |  |  |  |  |  |  |  |  |  |  |  |  |  |
| 3. Openness/Intellect (Higher Meat Consumer) | -.33 | [-.47, -.17] | -.13 | [-.30, .04] |  |  |  |  |  |  |  |  |  |  |  |
| 4. Openness/Intellect (Lower Meat Consumer) | -.18 | [-.33, -.01] | -.10 | [-.27, .07] | .44 | [.30, .57] |  |  |  |  |  |  |  |  |  |
| 5. Openness (Higher Meat Consumer) | -.26 | [-.41, -.10] | -.06 | [-.22, .11] | .83 | [.76, .87] | .44 | [.29, .56] |  |  |  |  |  |  |  |
| 6. Openness (Lower Meat Consumer) | -.17 | [-.33, -.01] | -.08 | [-.25, .09] | .45 | [.31, .58] | .85 | [.80, .89] | .57 | [.45, .68] |  |  |  |  |  |
| 7. Intellect (Higher Meat Consumer) | -.26 | [-.41, -.10] | -.16 | [-.32, .01] | .77 | [.69, .83] | .26 | [.10, .41] | .27 | [.10, .42] | .12 | [-.05, .28] |  |  |  |
| 8. Intellect (Lower Meat Consumer) | -.10 | [-.27, .07] | -.08 | [-.25, .09] | .24 | [.08, .40] | .76 | [.68, .82] | .08 | [-.09, .25] | .31 | [.15, .46] | .32 | [.16, .47] |  |
| Mean | 1.1 |  | -.9 |  | 3.7 |  | 3.7 |  | 3.6 |  | 3.5 |  | 3.9 |  | 3.8 |
| SD | 1.6 |  | 1.5 |  | .4 |  | .4 |  | .5 |  | .6 |  | .5 |  | .5 |
| Min | -2.1 |  | -2.5 |  | 3.0 |  | 2.6 |  | 2.0 |  | 2.0 |  | 2.6 |  | 2.6 |
| Max | 4.8 |  | 2.9 |  | 4.6 |  | 4.8 |  | 4.9 |  | 4.7 |  | 4.9 |  | 5.0 |

*Note.* 95% confidence intervals are reported in square brackets; “Higher Meat Consumer” refers to the partner who consumed more meat; “Lower Meat Consumer” refers to the partner who consumes less meat; Meat consumption is comprised of general and shared meat consumption; Personality is comprised of self and informant-reports.

## Table S7

*Pairwise Pearson Correlations and Descriptive Statistics for Meat Consumption and Traits Within the Agreeableness Domain Split by Measurement Method.*

|  | 1. |  | 2. |  | 3. |  | 4. |  | 5. |  | 6. |  | 7. |  | 8. |  | 9. |  | 10. |  | 11. |  | 12. |
| --- | --- | --- | --- | --- | --- | --- | --- | --- | --- | --- | --- | --- | --- | --- | --- | --- | --- | --- | --- | --- | --- | --- | --- |
| 1. Combined Meat |  |  |  |  |  |  |  |  |  |  |  |  |  |  |  |  |  |  |  |  |  |  |  |
| 2. General Meat | .93 | [.91, .94] |  |  |  |  |  |  |  |  |  |  |  |  |  |  |  |  |  |  |  |  |  |
| 3. Shared Meat | .93 | [.91, .94] | .72 | [.66, .77] |  |  |  |  |  |  |  |  |  |  |  |  |  |  |  |  |  |  |  |
| 4. Agreeableness (aggregated) | -.14 | [-.25, -.02] | -.13 | [-.24, -.01] | -.13 | [-.24, -.01] |  |  |  |  |  |  |  |  |  |  |  |  |  |  |  |  |  |
| 5. Agreeableness (self-reported) | -.25 | [-.35, -.13] | -.26 | [-.37, -.14] | -.20 | [-.31, -.08] | .76 | [.70, .80] |  |  |  |  |  |  |  |  |  |  |  |  |  |  |  |
| 6. Agreeableness (partner-reported) | .02 | [-.10, .14] | .05 | [-.07, .16] | -.01 | [-.13, .11] | .80 | [.75, .84] | .22 | [.10, .33] |  |  |  |  |  |  |  |  |  |  |  |  |  |
| 7. Compassion (aggregated) | -.18 | [-.30, -.07] | -.16 | [-.27, -.04] | -.18 | [-.29, -.06] | .84 | [.81, .87] | .68 | [.61, .74] | .64 | [.56, .70] |  |  |  |  |  |  |  |  |  |  |  |
| 8. Compassion (self-reported) | -.29 | [-.39, -.18] | -.29 | [-.40, -.18] | -.24 | [-.35, -.13] | .59 | [.51, .66] | .86 | [.83, .89] | .10 | [-.02, .21] | .74 | [.68, .79] |  |  |  |  |  |  |  |  |  |
| 9. Compassion (partner-reported) | .03 | [-.09, .14] | .07 | [-.05, .18] | -.02 | [-.14, .10] | .64 | [.56, .70] | .12 | [.002, .24] | .84 | [.80, .87] | .72 | [.66, .77] | .06 | [-.06, .18] |  |  |  |  |  |  |  |
| 10. Politeness (aggregated) | -.05 | [-.16, .07] | -.05 | [-.17, .07] | -.03 | [-.15, .09] | .83 | [.79, .87] | .59 | [.50, .66] | .71 | [.64, .76] | .40 | [.30, .50] | .25 | [.13, .36] | .34 | [.23, .44] |  |  |  |  |  |
| 11. Politeness (self-reported) | -.09 | [-.21, .03] | -.10 | [-.22, .01] | -.06 | [-.18, .06] | .65 | [.58, .72] | .76 | [.71, .81] | .28 | [.17, .39] | .33 | [.22, .43] | .33 | [.22, .43] | .15 | [.03, .26] | .78 | [.72, .82] |  |  |  |
| 12. Politeness (partner-reported) | .01 | [-.11, .13] | .01 | [-.11, .13] | .003 | [-.12, .12] | .70 | [.63, .76] | .24 | [.13, .35] | .82 | [.78, .86] | .33 | [.22, .43] | .10 | [-.02, .21] | .39 | [.28, .49] | .85 | [.81, .88] | .32 | [.21, .43] |  |
| Mean | .1 |  | 5.4 |  | .3 |  | 4.0 |  | 3.9 |  | 4.0 |  | 4.1 |  | 4.2 |  | 4.1 |  | 3.8 |  | 3.7 |  | 3.9 |
| SD | 1.8 |  | 2.6 |  | .3 |  | .3 |  | .4 |  | .5 |  | .4 |  | .6 |  | .6 |  | .4 |  | .5 |  | .5 |
| Min | -2.5 |  | 2.0 |  | 0 |  | 2.8 |  | 2.7 |  | 2.3 |  | 2.8 |  | 2.6 |  | 1.8 |  | 2.4 |  | 2.4 |  | 2.2 |
| Max | 4.8 |  | 14.0 |  | 1.0 |  | 4.7 |  | 4.7 |  | 4.9 |  | 5.0 |  | 5.0 |  | 5.0 |  | 4.6 |  | 4.9 |  | 4.9 |

*Note.* 95% Confidence Intervals are reported in square brackets; Combined meat consumption is comprised of general and shared meat consumption; General meat consumption is measured using the baseline survey; Shared meat consumption is measured using daily diaries.

## Table S8

*Pairwise Pearson Correlations and Descriptive Statistics for Meat Consumption and Traits Within the Agreeableness Domain Split by Level of Meat Consumption.*

|  | 1. |  | 2. |  | 3. |  | 4. |  | 5. |  | 6. |  | 7. |  | 8. |
| --- | --- | --- | --- | --- | --- | --- | --- | --- | --- | --- | --- | --- | --- | --- | --- |
| 1. Meat Consumption (Higher Meat Consumer) |  |  |  |  |  |  |  |  |  |  |  |  |  |  |  |
| 2. Meat Consumption (Lower Meat Consumer) | .61 | [.49, .71] |  |  |  |  |  |  |  |  |  |  |  |  |  |
| 3. Agreeableness (Higher Meat Consumer) | -.14 | [-.30, .03] | .00 | [-.17, .17] |  |  |  |  |  |  |  |  |  |  |  |
| 4. Agreeableness (Lower Meat Consumer) | -.03 | [-.20, .14] | -.10 | [-.26, .07] | .27 | [.11, .42] |  |  |  |  |  |  |  |  |  |
| 5. Compassion (Higher Meat Consumer) | -.19 | [-.35, -.03] | -.06 | [-.23, .10] | .86 | [.81, .90] | .13 | [-.04, .30] |  |  |  |  |  |  |  |
| 6. Compassion (Lower Meat Consumer) | -.04 | [-.21, .13] | -.12 | [-.28, .05] | .20 | [.03, .36] | .82 | [.76, .87] | .21 | [.05, .37] |  |  |  |  |  |
| 7. Politeness (Higher Meat Consumer) | -.04 | [-.21, .13] | .07 | [-.10, .24] | .84 | [.78, .88] | .33 | [.17, .47] | .45 | [.30, .57] | .13 | [-.04, .29] |  |  |  |
| 8. Politeness (Lower Meat Consumer) | -.02 | [-.18, .15] | -.05 | [-.21, .12] | .24 | [.08, .39] | .83 | [.77, .87] | .01 | [-.16, .18] | .36 | [.21, .50] | .42 | [.27, .55] |  |
| Mean | 1.1 |  | -.9 |  | 3.9 |  | 4.0 |  | 4.1 |  | 4.2 |  | 3.8 |  | 3.8 |
| SD | 1.6 |  | 1.5 |  | .4 |  | .3 |  | .4 |  | .4 |  | .4 |  | .4 |
| Min | -2.1 |  | -2.5 |  | 2.8 |  | 2.9 |  | 2.9 |  | 2.8 |  | 2.6 |  | 2.4 |
| Max | 4.8 |  | 2.9 |  | 4.6 |  | 4.7 |  | 5.0 |  | 5.0 |  | 4.6 |  | 4.6 |

*Note.* 95% confidence intervals are reported in square brackets; “Higher Meat Consumer” refers to the partner who consumed more meat; “Lower Meat Consumer” refers to the partner who consumes less meat.

## Table S9

*Pairwise Pearson Correlations and Descriptive Statistics for Meat Consumption and Traits Within the Neuroticism Domain Split by Measurement Method.*

|  | 1. |  | 2. |  | 3. |  | 4. |  | 5. |  | 6. |  | 7. |  | 8. |  | 9. |  | 10. |  | 11. |  | 12. |
| --- | --- | --- | --- | --- | --- | --- | --- | --- | --- | --- | --- | --- | --- | --- | --- | --- | --- | --- | --- | --- | --- | --- | --- |
| 1. Combined Meat |  |  |  |  |  |  |  |  |  |  |  |  |  |  |  |  |  |  |  |  |  |  |  |
| 2. General Meat | .93 | [.91, .94] |  |  |  |  |  |  |  |  |  |  |  |  |  |  |  |  |  |  |  |  |  |
| 3. Shared Meat | .93 | [.91, .94] | .72 | [.66, .77] |  |  |  |  |  |  |  |  |  |  |  |  |  |  |  |  |  |  |  |
| 4. Neuroticism (aggregated) | .01 | [-.11, .13] | .03 | [-.09, .15] | -.003 | [-.12, .12] |  |  |  |  |  |  |  |  |  |  |  |  |  |  |  |  |  |
| 5. Neuroticism (self-reported) | -.21 | [-.32, -.10] | -.23 | [-.34, -.11] | -.17 | [-.28, -.05] | .62 | [.54, .69] |  |  |  |  |  |  |  |  |  |  |  |  |  |  |  |
| 6. Neuroticism (partner-reported) | .21 | [.09, .32] | .24 | [.13, .35] | .15 | [.03, .26] | .71 | [.65, .77] | -.11 | [-.22, .01] |  |  |  |  |  |  |  |  |  |  |  |  |  |
| 7. Withdrawal (aggregated) | -.03 | [-.15, .09] | -.02 | [-.14, .10] | -.04 | [-.16, .08] | .90 | [.87, .92] | .59 | [.51, .66] | .61 | [.53, .68] |  |  |  |  |  |  |  |  |  |  |  |
| 8. Withdrawal (self-reported) | -.25 | [-.36, -.14] | -.26 | [-.37, -.15] | -.20 | [-.32, -.09] | .57 | [.48, .64] | .92 | [.90, .94] | -.10 | [-.22, .01] | .65 | [.58, .72] |  |  |  |  |  |  |  |  |  |
| 9. Withdrawal (partner-reported) | .19 | [.08, .31] | .22 | [.10, .33] | .14 | [.02, .25] | .66 | [.59, .72] | -.07 | [-.19, .05] | .90 | [.88, .92] | .71 | [.65, .77] | -.06 | [-.18, .06] |  |  |  |  |  |  |  |
| 10. Volatility (aggregated) | .06 | [-.06, .17] | .07 | [-.05, .19] | .03 | [-.09, .15] | .89 | [.87, .92] | .52 | [.43, .61] | .66 | [.59, .73] | .61 | [.53, .68] | .36 | [.25, .46] | .47 | [.37, .56] |  |  |  |  |  |
| 11. Volatility (self-reported) | -.13 | [-.25, -.02] | -.15 | [-.27, -.03] | -.10 | [-.21, .02] | .57 | [.48, .64] | .91 | [.89, .93] | -.09 | [-.21, .03] | .42 | [.32, .51] | .67 | [.60, .73] | -.07 | [-.18, .05] | .60 | [.52, .67] |  |  |  |
| 12. Volatility (partner-reported) | .18 | [.07, .30] | .22 | [.10, .33] | .12 | [.01, .24] | .63 | [.55, .69] | -.13 | [-.24, -.01] | .91 | [.89, .93] | .40 | [.29, .49] | -.12 | [-.24, -.01] | .64 | [.56, .71] | .73 | [.67, .78] | -.10 | [-.22, .02] |  |
| Mean | .1 |  | 5.4 |  | .3 |  | 2.6 |  | 2.6 |  | 2.6 |  | 2.8 |  | 2.7 |  | 2.8 |  | 2.5 |  | 2.5 |  | 2.4 |
| SD | 1.8 |  | 2.6 |  | .3 |  | .5 |  | .7 |  | .7 |  | .5 |  | .7 |  | .8 |  | .5 |  | .7 |  | .8 |
| Min | -2.5 |  | 2.0 |  | 0 |  | 1.2 |  | 1.1 |  | 1.0 |  | 1.2 |  | 1.1 |  | 1.0 |  | 1.0 |  | 1.0 |  | 1.0 |
| Max | 4.8 |  | 14.0 |  | 1.0 |  | 3.9 |  | 4.6 |  | 4.9 |  | 4.2 |  | 4.9 |  | 4.8 |  | 4.0 |  | 4.4 |  | 5.0 |

*Note.* 95% Confidence Intervals are reported in square brackets; Combined meat consumption is comprised of general and shared meat consumption; General meat consumption is measured using the baseline survey; Shared meat consumption is measured using daily diaries.

## Table S10

*Pairwise Pearson Correlations and Descriptive Statistics for Meat Consumption and Traits Within the Neuroticism Domain Split by Level of Meat Consumption.*

|  | 1. |  | 2. |  | 3. |  | 4. |  | 5. |  | 6. |  | 7. |  | 8. |
| --- | --- | --- | --- | --- | --- | --- | --- | --- | --- | --- | --- | --- | --- | --- | --- |
| 1. Meat Consumption (Higher Meat Consumer) |  |  |  |  |  |  |  |  |  |  |  |  |  |  |  |
| 2. Meat Consumption (Lower Meat Consumer) | .61 | [.49, .71] |  |  |  |  |  |  |  |  |  |  |  |  |  |
| 3. Neuroticism (Higher Meat Consumer) | .06 | [-.11, .22] | -.13 | [-.29, .04] |  |  |  |  |  |  |  |  |  |  |  |
| 4. Neuroticism (Lower Meat Consumer) | -.07 | [-.24, .10] | -.04 | [-.21, .13] | .42 | [.27, .55] |  |  |  |  |  |  |  |  |  |
| 5. Withdrawal (Higher Meat Consumer) | -.01 | [-.18, .16] | -.16 | [-.32, .01] | .89 | [.85, .92] | .39 | [.24, .53] |  |  |  |  |  |  |  |
| 6. Withdrawal (Lower Meat Consumer) | -.10 | [-.26, .07] | -.09 | [-.25, .08] | .40 | [.25, .53] | .91 | [.87, .93] | .45 | [.31, .58] |  |  |  |  |  |
| 7. Volatility (Higher Meat Consumer) | .11 | [-.06, .28] | -.07 | [-.24, .10] | .90 | [.86, .93] | .36 | [.21, .50] | .60 | [.48, .70] | .27 | [.10, .42] |  |  |  |
| 8. Volatility (Lower Meat Consumer) | -.03 | [-.20, .14] | .02 | [-.15, .19] | .36 | [.20, .50] | .89 | [.85, .92] | .25 | [.08, .40] | .62 | [.50, .71] | .39 | [.24, .52] |  |
| Mean | 1.1 |  | -.9 |  | 2.6 |  | 2.6 |  | 2.8 |  | 2.7 |  | 2.5 |  | 2.5 |
| SD | 1.6 |  | 1.5 |  | .4 |  | .5 |  | .5 |  | .6 |  | .5 |  | .5 |
| Min | -2.1 |  | -2.5 |  | 1.4 |  | 1.2 |  | 1.3 |  | 1.2 |  | 1.0 |  | 1.1 |
| Max | 4.8 |  | 2.9 |  | 3.9 |  | 3.9 |  | 4.0 |  | 4.2 |  | 4.0 |  | 4.0 |

*Note.* 95% confidence intervals are reported in square brackets; “Higher Meat Consumer” refers to the partner who consumed more meat; “Lower Meat Consumer” refers to the partner who consumes less meat; Meat consumption is comprised of general and shared meat consumption; Personality is comprised of self and informant-reports.

## Table S11

*Pairwise Pearson Correlations and Descriptive Statistics for Meat Consumption and Traits Within the Extraversion Domain Split by Measurement Method.*

|  | 1. |  | 2. |  | 3. |  | 4. |  | 5. |  | 6. |  | 7. |  | 8. |  | 9. |  | 10. |  | 11. |  | 12. |
| --- | --- | --- | --- | --- | --- | --- | --- | --- | --- | --- | --- | --- | --- | --- | --- | --- | --- | --- | --- | --- | --- | --- | --- |
| 1. Combined Meat |  |  |  |  |  |  |  |  |  |  |  |  |  |  |  |  |  |  |  |  |  |  |  |
| 2. General Meat | .93 | [.91, .94] |  |  |  |  |  |  |  |  |  |  |  |  |  |  |  |  |  |  |  |  |  |
| 3. Shared Meat | .93 | [.91, .94] | .72 | [.66, .77] |  |  |  |  |  |  |  |  |  |  |  |  |  |  |  |  |  |  |  |
| 4. Extraversion (aggregated) | -.08 | [-.20, .04] | -.07 | [-.18, .05] | -.09 | [-.20, .03] |  |  |  |  |  |  |  |  |  |  |  |  |  |  |  |  |  |
| 5. Extraversion (self-reported) | .03 | [-.09, .15] | .05 | [-.07, .16] | .01 | [-.11, .12] | .72 | [.66, .77] |  |  |  |  |  |  |  |  |  |  |  |  |  |  |  |
| 6. Extraversion (partner-reported) | -.15 | [-.26, -.03] | -.14 | [-.25, -.02] | -.13 | [-.25, -.01] | .75 | [.70, .80] | .08 | [-.04, .20] |  |  |  |  |  |  |  |  |  |  |  |  |  |
| 7. Enthusiasm (aggregated) | -.07 | [-.19, .05] | -.06 | [-.17, .06] | -.08 | [-.19, .04] | .88 | [.85, .90] | .61 | [.53, .68] | .68 | [.61, .74] |  |  |  |  |  |  |  |  |  |  |  |
| 8. Enthusiasm (self-reported) | -.01 | [-.13, .10] | -1e-04 | [-.12, .12] | -.03 | [-.15, .09] | .67 | [.59, .73] | .85 | [.81, .88] | .15 | [.03, .26] | .73 | [.67, .78] |  |  |  |  |  |  |  |  |  |
| 9. Enthusiasm (partner-reported) | -.09 | [-.21, .03] | -.08 | [-.20, .04] | -.09 | [-.20, .03] | .66 | [.58, .72] | .09 | [-.03, .21] | .85 | [.82, .88] | .77 | [.72, .82] | .14 | [.02, .25] |  |  |  |  |  |  |  |
| 10. Assertiveness (aggregated) | -.07 | [-.19, .05] | -.06 | [-.18, .06] | -.08 | [-.19, .04] | .84 | [.81, .88] | .63 | [.56, .70] | .61 | [.53, .68] | .48 | [.38, .57] | .39 | [.29, .49] | .33 | [.22, .44] |  |  |  |  |  |
| 11. Assertiveness (self-reported) | .06 | [-.06, .18] | .08 | [-.04, .19] | .04 | [-.08, .16] | .55 | [.46, .63] | .85 | [.81, .88] | -.01 | [-.13, .11] | .29 | [.18, .40] | .44 | [.34, .53] | .02 | [-.10, .14] | .68 | [.61, .74] |  |  |  |
| 12. Assertiveness (partner-reported) | -.16 | [-.27, -.04] | -.15 | [-.27, -.03] | -.14 | [-.25, -.02] | .62 | [.54, .69] | .05 | [-.07, .17] | .84 | [.80, .87] | .38 | [.27, .47] | .12 | [-.002, .23] | .44 | [.34, .53] | .71 | [.64, .76] | -.03 | [-.15, .08] |  |
| Mean | .1 |  | 5.4 |  | .3 |  | 3.6 |  | 3.6 |  | 3.6 |  | 3.8 |  | 3.7 |  | 3.8 |  | 3.4 |  | 3.4 |  | 3.4 |
| SD | 1.8 |  | 2.6 |  | .3 |  | .4 |  | .5 |  | .5 |  | .5 |  | .6 |  | .6 |  | .4 |  | .6 |  | .6 |
| Min | -2.5 |  | 2.0 |  | 0 |  | 2.5 |  | 1.9 |  | 1.9 |  | 2.2 |  | 1.9 |  | 1.8 |  | 2.2 |  | 1.6 |  | 1.4 |
| Max | 4.8 |  | 14.0 |  | 1.0 |  | 4.6 |  | 4.7 |  | 4.8 |  | 4.9 |  | 5.0 |  | 5.0 |  | 4.5 |  | 4.9 |  | 4.8 |

*Note.* 95% Confidence Intervals are reported in square brackets; Combined meat consumption is comprised of general and shared meat consumption; General meat consumption is measured using the baseline survey; Shared meat consumption is measured using daily diaries.

**Table S12**

*Pairwise Pearson Correlations and Descriptive Statistics for Meat Consumption and Traits Within the Extraversion Domain Split by Level of Meat Consumption.*

|  | 1. |  | 2. |  | 3. |  | 4. |  | 5. |  | 6. |  | 7. |  | 8. |
| --- | --- | --- | --- | --- | --- | --- | --- | --- | --- | --- | --- | --- | --- | --- | --- |
| 1. Meat Consumption (Higher Meat Consumer) |  |  |  |  |  |  |  |  |  |  |  |  |  |  |  |
| 2. Meat Consumption (Lower Meat Consumer) | .61 | [.49, .71] |  |  |  |  |  |  |  |  |  |  |  |  |  |
| 3. Extraversion (Higher Meat Consumer) | -.12 | [-.28, .05] | .06 | [-.11, .23] |  |  |  |  |  |  |  |  |  |  |  |
| 4. Extraversion (Lower Meat Consumer) | .07 | [-.10, .24] | -.01 | [-.18, .16] | .29 | [.13, .44] |  |  |  |  |  |  |  |  |  |
| 5. Enthusiasm (Higher Meat Consumer) | -.07 | [-.24, .10] | .05 | [-.11, .22] | .89 | [.84, .92] | .23 | [.07, .39] |  |  |  |  |  |  |  |
| 6. Enthusiasm (Lower Meat Consumer) | .06 | [-.11, .23] | -.06 | [-.23, .10] | .24 | [.07, .39] | .87 | [.82, .90] | .26 | [.09, .41] |  |  |  |  |  |
| 7. Assertiveness (Higher Meat Consumer) | -.13 | [-.29, .04] | .05 | [-.12, .22] | .86 | [.81, .90] | .28 | [.12, .43] | .53 | [.39, .64] | .16 | [-.01, .32] |  |  |  |
| 8. Assertiveness (Lower Meat Consumer) | .07 | [-.10, .23] | .05 | [-.12, .22] | .26 | [.09, .41] | .82 | [.76, .87] | .13 | [-.04, .29] | .43 | [.29, .56] | .33 | [.17, .47] |  |
| Mean | 1.1 |  | -.9 |  | 3.6 |  | 3.6 |  | 3.8 |  | 3.8 |  | 3.4 |  | 3.4 |
| SD | 1.6 |  | 1.5 |  | .4 |  | .4 |  | .5 |  | .5 |  | .4 |  | .4 |
| Min | -2.1 |  | -2.5 |  | 2.6 |  | 2.5 |  | 2.5 |  | 2.2 |  | 2.2 |  | 2.4 |
| Max | 4.8 |  | 2.9 |  | 4.6 |  | 4.4 |  | 4.9 |  | 4.9 |  | 4.5 |  | 4.4 |

*Note.* 95% confidence intervals are reported in square brackets; “Higher Meat Consumer” refers to the partner who consumed more meat; “Lower Meat Consumer” refers to the partner who consumes less meat; Meat consumption is comprised of general and shared meat consumption; Personality is comprised of self and informant-reports.

## Table S12

*Pairwise Pearson Correlations and Descriptive Statistics for Meat Consumption and Traits Within the Conscientiousness Domain Split by Measurement Method.*

|  | 1. |  | 2. |  | 3. |  | 4. |  | 5. |  | 6. |  | 7. |  | 8. |  | 9. |  | 10. |  | 11. |  | 12. |
| --- | --- | --- | --- | --- | --- | --- | --- | --- | --- | --- | --- | --- | --- | --- | --- | --- | --- | --- | --- | --- | --- | --- | --- |
| 1. Combined Meat |  |  |  |  |  |  |  |  |  |  |  |  |  |  |  |  |  |  |  |  |  |  |  |
| 2. General Meat | .93 | [.91, .94] |  |  |  |  |  |  |  |  |  |  |  |  |  |  |  |  |  |  |  |  |  |
| 3. Shared Meat | .93 | [.91, .94] | .72 | [.66, .77] |  |  |  |  |  |  |  |  |  |  |  |  |  |  |  |  |  |  |  |
| 4. Conscientiousness (aggregated) | .01 | [-.10, .13] | -.02 | [-.13, .10] | .04 | [-.08, .16] |  |  |  |  |  |  |  |  |  |  |  |  |  |  |  |  |  |
| 5. Conscientiousness (self-reported) | -.04 | [-.16, .08] | -.06 | [-.18, .06] | -.01 | [-.13, .11] | .66 | [.58, .72] |  |  |  |  |  |  |  |  |  |  |  |  |  |  |  |
| 6. Conscientiousness (partner-reported) | .06 | [-.06, .17] | .03 | [-.09, .15] | .07 | [-.05, .19] | .71 | [.65, .77] | -.06 | [-.18, .06] |  |  |  |  |  |  |  |  |  |  |  |  |  |
| 7. Orderliness (aggregated) | .04 | [-.08, .15] | .005 | [-.11, .12] | .06 | [-.06, .18] | .83 | [.79, .86] | .50 | [.41, .59] | .63 | [.55, .70] |  |  |  |  |  |  |  |  |  |  |  |
| 8. Orderliness (self-reported) | -.04 | [-.16, .08] | -.06 | [-.18, .06] | -.01 | [-.13, .11] | .52 | [.42, .60] | .85 | [.82, .88] | -.11 | [-.22, .01] | .61 | [.53, .68] |  |  |  |  |  |  |  |  |  |
| 9. Orderliness (partner-reported) | .08 | [-.04, .20] | .06 | [-.06, .17] | .09 | [-.03, .21] | .60 | [.52, .67] | -.10 | [-.22, .02] | .88 | [.86, .91] | .73 | [.67, .78] | -.09 | [-.21, .03] |  |  |  |  |  |  |  |
| 10. Industriousness (aggregated) | -.01 | [-.13, .11] | -.03 | [-.15, .09] | .01 | [-.11, .13] | .84 | [.80, .87] | .59 | [.51, .66] | .57 | [.48, .64] | .39 | [.29, .49] | .26 | [.14, .36] | .27 | [.16, .38] |  |  |  |  |  |
| 11. Industriousness (self-reported) | -.03 | [-.15, .09] | -.04 | [-.16, .08] | -.01 | [-.13, .11] | .61 | [.53, .68] | .86 | [.83, .89] | .01 | [-.11, .12] | .26 | [.14, .37] | .47 | [.38, .56] | -.08 | [-.20, .04] | .75 | [.69, .80] |  |  |  |
| 12. Industriousness (partner-reported) | .01 | [-.11, .13] | -.003 | [-.12, .12] | .03 | [-.09, .15] | .64 | [.57, .71] | .01 | [-.11, .13] | .84 | [.81, .88] | .33 | [.22, .43] | -.10 | [-.21, .02] | .50 | [.40, .58] | .74 | [.68, .79] | .11 | [-.01, .22] |  |
| Mean | .1 |  | 5.4 |  | .3 |  | 3.5 |  | 3.4 |  | 3.5 |  | 3.4 |  | 3.4 |  | 3.3 |  | 3.6 |  | 3.5 |  | 3.7 |
| SD | 1.8 |  | 2.6 |  | .3 |  | .4 |  | .6 |  | .6 |  | .5 |  | .6 |  | .7 |  | .5 |  | .7 |  | .6 |
| Min | -2.5 |  | 2.0 |  | 0 |  | 2.1 |  | 1.8 |  | 1.6 |  | 2.3 |  | 1.5 |  | 1.4 |  | 2.0 |  | 1.5 |  | 1.8 |
| Max | 4.8 |  | 14.0 |  | 1.0 |  | 4.6 |  | 4.8 |  | 4.9 |  | 4.7 |  | 4.9 |  | 5.0 |  | 5.0 |  | 5.0 |  | 5.0 |

*Note.* 95% Confidence Intervals are reported in square brackets; Combined meat consumption is comprised of general and shared meat consumption; General meat consumption is measured using the baseline survey; Shared meat consumption is measured using daily diaries.

## Table S13

*Pairwise Pearson Correlations and Descriptive Statistics for Meat Consumption and Traits Within the Conscientiousness Domain Split by Level of Meat Consumption.*

|  | 1. |  | 2. |  | 3. |  | 4. |  | 5. |  | 6. |  | 7. |  | 8. |
| --- | --- | --- | --- | --- | --- | --- | --- | --- | --- | --- | --- | --- | --- | --- | --- |
| 1. Meat Consumption (Higher Meat Consumer) |  |  |  |  |  |  |  |  |  |  |  |  |  |  |  |
| 2. Meat Consumption (Lower Meat Consumer) | .61 | [.49, .71] |  |  |  |  |  |  |  |  |  |  |  |  |  |
| 3. Conscientiousness (Higher Meat Consumer) | -.01 | [-.18, .16] | .05 | [-.12, .22] |  |  |  |  |  |  |  |  |  |  |  |
| 4. Conscientiousness (Lower Meat Consumer) | .03 | [-.14, .20] | .02 | [-.15, .19] | .51 | [.37, .62] |  |  |  |  |  |  |  |  |  |
| 5. Orderliness (Higher Meat Consumer) | .02 | [-.15, .19] | .05 | [-.12, .21] | .81 | [.75, .86] | .47 | [.32, .59] |  |  |  |  |  |  |  |
| 6. Orderliness (Lower Meat Consumer) | .04 | [-.13, .21] | .02 | [-.14, .19] | .36 | [.20, .49] | .84 | [.78, .88] | .42 | [.28, .55] |  |  |  |  |  |
| 7. Industriousness (Higher Meat Consumer) | -.04 | [-.20, .13] | .04 | [-.13, .21] | .84 | [.78, .88] | .37 | [.22, .51] | .36 | [.20, .50] | .17 | [.002, .33] |  |  |  |
| 8. Industriousness (Lower Meat Consumer) | .01 | [-.15, .18] | .005 | [-.16, .17] | .50 | [.36, .62] | .85 | [.79, .89] | .36 | [.21, .50] | .42 | [.28, .55] | .46 | [.31, .58] |  |
| Mean | 1.1 |  | -.9 |  | 3.5 |  | 3.5 |  | 3.4 |  | 3.4 |  | 3.6 |  | 3.6 |
| SD | 1.6 |  | 1.5 |  | .4 |  | .4 |  | .4 |  | .5 |  | .5 |  | .5 |
| Min | -2.1 |  | -2.5 |  | 2.1 |  | 2.2 |  | 2.4 |  | 2.3 |  | 2.0 |  | 2.0 |
| Max | 4.8 |  | 2.9 |  | 4.6 |  | 4.5 |  | 4.7 |  | 4.4 |  | 4.8 |  | 5.0 |

*Note.* 95% confidence intervals are reported in square brackets; “Higher Meat Consumer” refers to the partner who consumed more meat; “Lower Meat Consumer” refers to the partner who consumes less meat; Meat consumption is comprised of general and shared meat consumption; Personality is comprised of self and informant-reports.

## Table S14

*Comparisons between Constrained (Indistinguishable) and Unconstrained (Distinguishable) Models.*

| Model | Trait | ΔChi-square | *p* | ΔAIC | ΔBIC |
| --- | --- | --- | --- | --- | --- |
| Combined Meat Consumption | Openness/Intellect | 38.81 | < .001 | 26.81 | 9.34 |
|  | Openness | 41.05 | < .001 | 29.05 | 11.57 |
|  | Intellect | 34.12 | < .001 | 22.12 | 4.64 |
|  | Agreeableness | 31.32 | < .001 | 19.32 | 1.85 |
|  | Politeness | 31.82 | < .001 | 19.82 | 2.35 |
|  | Compassion | 32.96 | < .001 | 20.96 | 3.48 |
|  | Conscientiousness | 32.02 | < .001 | 20.02 | 2.54 |
|  | Industriousness | 31.15 | < .001 | 19.15 | 1.67 |
|  | Orderliness | 32.84 | < .001 | 20.84 | 3.37 |
|  | Neuroticism | 33.11 | < .001 | 21.11 | 3.64 |
|  | Withdrawal | 35.75 | < .001 | 23.75 | 6.27 |
|  | Volatility | 31.29 | < .001 | 19.29 | 1.82 |
|  | Extraversion | 29.16 | < .001 | 17.16 | -0.32 |
|  | Assertiveness | 31.79 | < .001 | 19.79 | 2.31 |
|  | Enthusiasm | 29.16 | < .001 | 17.16 | -0.32 |
| General Meat Consumption | Openness/Intellect | 55.47 | < .001 | 43.47 | 25.99 |
|  | Openness | 56.57 | < .001 | 44.57 | 27.09 |
|  | Intellect | 53.82 | < .001 | 41.82 | 24.34 |
|  | Agreeableness | 48.43 | < .001 | 36.43 | 18.96 |
|  | Politeness | 48.15 | < .001 | 36.15 | 18.67 |
|  | Compassion | 50.56 | < .001 | 38.56 | 21.09 |
|  | Conscientiousness | 49.63 | < .001 | 37.63 | 20.15 |
|  | Industriousness | 48.62 | < .001 | 36.62 | 19.14 |
|  | Orderliness | 50.46 | < .001 | 38.46 | 20.98 |
|  | Neuroticism | 50.37 | < .001 | 38.37 | 20.9 |
|  | Withdrawal | 53.82 | < .001 | 41.82 | 24.34 |
|  | Volatility | 48.09 | < .001 | 36.09 | 18.61 |
|  | Extraversion | 47.65 | < .001 | 35.65 | 18.18 |
|  | Assertiveness | 49.87 | < .001 | 37.87 | 20.39 |
|  | Enthusiasm | 47.05 | < .001 | 35.05 | 17.58 |
| Shared Meat Consumption | Openness/Intellect | 19.73 | .003 | 7.73 | -9.75 |
|  | Openness | 22.02 | .001 | 10.02 | -7.45 |
|  | Intellect | 16.05 | .013 | 4.05 | -13.43 |
|  | Agreeableness | 16.62 | .011 | 4.62 | -12.85 |
|  | Politeness | 17.91 | .006 | 5.91 | -11.56 |
|  | Compassion | 17.12 | .009 | 5.12 | -12.35 |
|  | Conscientiousness | 16.66 | .011 | 4.66 | -12.82 |
|  | Industriousness | 15.52 | .017 | 3.52 | -13.95 |
|  | Orderliness | 17.01 | .009 | 5.01 | -12.47 |
|  | Neuroticism | 17.4 | .008 | 5.4 | -12.07 |
|  | Withdrawal | 19.45 | .003 | 7.45 | -10.03 |
|  | Volatility | 16.58 | .011 | 4.58 | -12.9 |
|  | Extraversion | 13.87 | .031 | 1.87 | -15.6 |
|  | Assertiveness | 16.18 | .013 | 4.18 | -13.3 |
|  | Enthusiasm | 14.26 | .027 | 2.26 | -15.21 |
| Self-Reported Personality | Openness/Intellect | 35.41 | < .001 | 23.41 | 5.93 |
|  | Openness | 40.02 | < .001 | 28.02 | 10.54 |
|  | Intellect | 34.1 | < .001 | 22.1 | 4.62 |
|  | Agreeableness | 51.26 | < .001 | 39.26 | 21.79 |
|  | Politeness | 38.64 | < .001 | 26.64 | 9.17 |
|  | Compassion | 49.43 | < .001 | 37.43 | 19.96 |
|  | Conscientiousness | 36.23 | < .001 | 24.23 | 6.75 |
|  | Industriousness | 33.24 | < .001 | 21.24 | 3.77 |
|  | Orderliness | 36.56 | < .001 | 24.56 | 7.09 |
|  | Neuroticism | 61.82 | < .001 | 49.82 | 32.34 |
|  | Withdrawal | 61.65 | < .001 | 49.65 | 32.18 |
|  | Volatility | 54.8 | < .001 | 42.8 | 25.32 |
|  | Extraversion | 32.51 | < .001 | 20.51 | 3.03 |
|  | Assertiveness | 35.06 | < .001 | 23.06 | 5.59 |
|  | Enthusiasm | 31.48 | < .001 | 19.48 | 2 |
| Partner-Reported Personality | Openness/Intellect | 37.98 | < .001 | 25.98 | 8.5 |
|  | Openness | 44.94 | < .001 | 32.94 | 15.46 |
|  | Intellect | 32.09 | < .001 | 20.09 | 2.62 |
|  | Agreeableness | 37.7 | < .001 | 25.7 | 8.23 |
|  | Politeness | 36.05 | < .001 | 24.05 | 6.57 |
|  | Compassion | 37.26 | < .001 | 25.26 | 7.79 |
|  | Conscientiousness | 38.58 | < .001 | 26.58 | 9.1 |
|  | Industriousness | 33.55 | < .001 | 21.55 | 4.07 |
|  | Orderliness | 38.51 | < .001 | 26.51 | 9.03 |
|  | Neuroticism | 50.7 | < .001 | 38.7 | 21.22 |
|  | Withdrawal | 55.34 | < .001 | 43.34 | 25.87 |
|  | Volatility | 42.86 | < .001 | 30.86 | 13.38 |
|  | Extraversion | 33.03 | < .001 | 21.03 | 3.55 |
|  | Assertiveness | 39.07 | < .001 | 27.07 | 9.59 |
|  | Enthusiasm | 31.34 | < .001 | 19.34 | 1.86 |
| Only Meat Consumption | Openness/Intellect | 39.97 | < .001 | 27.97 | 10.49 |
|  | Openness | 41.93 | < .001 | 29.93 | 12.45 |
|  | Intellect | 34.46 | < .001 | 22.46 | 4.98 |
|  | Agreeableness | 31.76 | < .001 | 19.76 | 2.29 |
|  | Politeness | 32.08 | < .001 | 20.08 | 2.61 |
|  | Compassion | 33.21 | < .001 | 21.21 | 3.74 |
|  | Conscientiousness | 31.54 | < .001 | 19.54 | 2.07 |
|  | Industriousness | 31.27 | < .001 | 19.27 | 1.79 |
|  | Orderliness | 31.81 | < .001 | 19.81 | 2.33 |
|  | Neuroticism | 32.49 | < .001 | 20.49 | 3.01 |
|  | Withdrawal | 35.06 | < .001 | 23.06 | 5.58 |
|  | Volatility | 31.54 | < .001 | 19.54 | 2.06 |
|  | Extraversion | 29.39 | < .001 | 17.39 | -0.09 |
|  | Assertiveness | 31.45 | < .001 | 19.45 | 1.97 |
|  | Enthusiasm | 29.09 | < .001 | 17.09 | -0.39 |
| Only Fish Consumption | Openness/Intellect | 13.29 | .039 | 1.29 | -16.18 |
|  | Openness | 14.04 | .029 | 2.04 | -15.44 |
|  | Intellect | 13.08 | .042 | 1.08 | -16.4 |
|  | Agreeableness | 12.54 | .051 | 0.54 | -16.93 |
|  | Politeness | 11.33 | .079 | -0.67 | -18.15 |
|  | Compassion | 14.83 | .022 | 2.83 | -14.64 |
|  | Conscientiousness | 12.5 | .052 | 0.5 | -16.98 |
|  | Industriousness | 10.81 | .094 | -1.19 | -18.67 |
|  | Orderliness | 12.8 | .046 | 0.8 | -16.67 |
|  | Neuroticism | 11.82 | .066 | -0.18 | -17.66 |
|  | Withdrawal | 14.99 | .020 | 2.99 | -14.48 |
|  | Volatility | 9.89 | .129 | -2.11 | -19.59 |
|  | Extraversion | 11.11 | .085 | -0.89 | -18.36 |
|  | Assertiveness | 12.35 | .055 | 0.35 | -17.13 |
|  | Enthusiasm | 10.41 | .108 | -1.59 | -19.07 |

*Note. “*ΔChi-square” = Chi-square of constrained model - Chi-square of unconstrained model; “ΔAIC” = AIC of constrained model - AIC of unconstrained model; “ΔBIC” = BIC of constrained model - BIC of unconstrained model; There was a 6 degrees of freedom difference for all comparisons.

## Table S15

*Standardized Actor and Partner Effects for Big Five Domain and Aspect Traits Split by Model.*

|  |  | Actor Effect (Higher Meat Consumer) | | | |  | Actor Effect (Lower Meat Consumer) | | | |  | Partner Effect (Higher Meat Consumer) | | | |  | Partner Effect (Lower Meat Consumer) | | | |  |  |  |
| --- | --- | --- | --- | --- | --- | --- | --- | --- | --- | --- | --- | --- | --- | --- | --- | --- | --- | --- | --- | --- | --- | --- | --- |
| Model | Trait | *ß* | *p* | Lower CI | Higher CI |  | *ß* | *p* | Lower CI | Higher CI |  | *ß* | *p* | Lower CI | Higher CI |  | *ß* | *p* | Lower CI | Higher CI |  | GFI | SRMR |
| Combined Meat Consumption | Openness/Intellect | **-.30** | **.001** | **-.47** | **-.13** |  | -.05 | .614 | -.23 | .14 |  | -.03 | .762 | -.20 | .15 |  | -.13 | .162 | -.31 | .05 |  | .998 | .063 |
|  | Openness | **-.24** | **.015** | **-.43** | **-.05** |  | -.07 | .505 | -.27 | .13 |  | -.04 | .711 | -.23 | .16 |  | -.02 | .823 | -.23 | .18 |  | .997 | .054 |
|  | Intellect | **-.24** | **.006** | **-.40** | **-.07** |  | -.03 | .712 | -.21 | .14 |  | < .001 | .996 | -.17 | .17 |  | **-.18** | **.042** | **-.35** | **-.01** |  | .997 | .075 |
|  | Agreeableness | -.13 | .146 | -.30 | .04 |  | -.10 | .266 | -.27 | .07 |  | -.02 | .852 | -.19 | .16 |  | .02 | .829 | -.15 | .19 |  | .998 | .066 |
|  | Politeness | -.03 | .751 | -.21 | .15 |  | -.09 | .352 | -.27 | .10 |  | -.02 | .795 | -.21 | .16 |  | .10 | .272 | -.08 | .29 |  | .997 | .066 |
|  | Compassion | **-.18** | **.038** | **-.34** | **-.01** |  | -.10 | .257 | -.27 | .07 |  | -.01 | .911 | -.18 | .16 |  | -.05 | .537 | -.22 | .12 |  | .998 | .061 |
|  | Conscientiousness | -.01 | .903 | -.21 | .18 |  | -.002 | .986 | -.20 | .19 |  | .05 | .594 | -.14 | .25 |  | .01 | .893 | -.18 | .21 |  | .997 | .086 |
|  | Industriousness | -.02 | .847 | -.21 | .17 |  | -.02 | .807 | -.21 | .16 |  | .05 | .629 | -.14 | .24 |  | .01 | .936 | -.18 | .20 |  | .995 | .100 |
|  | Orderliness | .01 | .943 | -.18 | .19 |  | .02 | .808 | -.16 | .21 |  | .04 | .681 | -.15 | .23 |  | .01 | .879 | -.17 | .20 |  | .996 | .066 |
|  | Neuroticism | .09 | .325 | -.09 | .28 |  | .03 | .773 | -.16 | .21 |  | -.12 | .204 | -.30 | .06 |  | -.11 | .261 | -.30 | .08 |  | .994 | .091 |
|  | Withdrawal | .01 | .909 | -.18 | .20 |  | -.02 | .842 | -.20 | .17 |  | -.12 | .221 | -.30 | .07 |  | -.11 | .257 | -.31 | .08 |  | .993 | .102 |
|  | Volatility | .15 | .112 | -.03 | .33 |  | .07 | .424 | -.11 | .25 |  | -.09 | .340 | -.27 | .09 |  | -.08 | .375 | -.27 | .10 |  | .993 | .073 |
|  | Extraversion | -.13 | .137 | -.31 | .04 |  | -.02 | .843 | -.19 | .16 |  | .11 | .211 | -.06 | .28 |  | .04 | .651 | -.14 | .22 |  | .998 | .068 |
|  | Assertiveness | -.15 | .091 | -.33 | .02 |  | .05 | .594 | -.13 | .23 |  | .11 | .202 | -.06 | .29 |  | .01 | .921 | -.17 | .19 |  | .997 | .070 |
|  | Enthusiasm | -.08 | .348 | -.26 | .09 |  | -.07 | .450 | -.24 | .11 |  | .08 | .368 | -.09 | .25 |  | .05 | .557 | -.12 | .23 |  | .997 | .064 |
| General Meat Consumption | Openness/Intellect | **-.35** | **< .001** | **-.52** | **-.19** |  | -.05 | .571 | -.23 | .13 |  | .03 | .748 | -.15 | .20 |  | -.14 | .129 | -.32 | .04 |  | .998 | .065 |
|  | Openness | **-.23** | **.018** | **-.42** | **-.04** |  | -.06 | .588 | -.26 | .15 |  | -.04 | .652 | -.24 | .15 |  | -.03 | .790 | -.23 | .17 |  | .997 | .056 |
|  | Intellect | **-.31** | **< .001** | **-.48** | **-.15** |  | -.06 | .491 | -.23 | .11 |  | .09 | .296 | -.08 | .26 |  | -.19 | .024 | -.36 | -.03 |  | .998 | .077 |
|  | Agreeableness | **-.18** | **.039** | **-.35** | **-.01** |  | -.02 | .823 | -.19 | .15 |  | .07 | .396 | -.10 | .25 |  | -.02 | .833 | -.19 | .15 |  | .998 | .068 |
|  | Politeness | -.13 | .171 | -.31 | .05 |  | -.02 | .850 | -.20 | .16 |  | .08 | .401 | -.11 | .26 |  | .04 | .704 | -.15 | .22 |  | .997 | .068 |
|  | Compassion | **-.18** | **.031** | **-.35** | **-.02** |  | -.02 | .773 | -.19 | .14 |  | .06 | .510 | -.11 | .22 |  | -.06 | .482 | -.23 | .11 |  | .998 | .063 |
|  | Conscientiousness | -.09 | .356 | -.29 | .10 |  | .02 | .803 | -.17 | .22 |  | .11 | .284 | -.09 | .30 |  | -.07 | .488 | -.26 | .13 |  | .997 | .088 |
|  | Industriousness | -.09 | .353 | -.28 | .10 |  | .01 | .888 | -.17 | .20 |  | .10 | .284 | -.09 | .29 |  | -.05 | .620 | -.24 | .14 |  | .996 | .101 |
|  | Orderliness | -.04 | .662 | -.22 | .14 |  | .02 | .872 | -.17 | .20 |  | .06 | .554 | -.13 | .24 |  | -.06 | .545 | -.24 | .13 |  | .997 | .068 |
|  | Neuroticism | .15 | .123 | -.04 | .33 |  | -.02 | .832 | -.20 | .16 |  | -.16 | .084 | -.34 | .02 |  | -.05 | .612 | -.24 | .14 |  | .994 | .092 |
|  | Withdrawal | .02 | .821 | -.17 | .21 |  | -.08 | .402 | -.26 | .11 |  | -.09 | .335 | -.28 | .09 |  | -.05 | .599 | -.25 | .14 |  | .994 | .103 |
|  | Volatility | **.22** | **.012** | **.05** | **.40** |  | .05 | .582 | -.13 | .23 |  | -.19 | .037 | -.36 | -.01 |  | -.04 | .689 | -.22 | .15 |  | .994 | .075 |
|  | Extraversion | -.14 | .106 | -.32 | .03 |  | .06 | .475 | -.11 | .24 |  | .16 | .058 | -.01 | .33 |  | .01 | .891 | -.16 | .19 |  | .998 | .070 |
|  | Assertiveness | -.11 | .215 | -.29 | .06 |  | .09 | .311 | -.08 | .27 |  | .10 | .240 | -.07 | .28 |  | .002 | .978 | -.17 | .18 |  | .997 | .071 |
|  | Enthusiasm | -.13 | .134 | -.30 | .04 |  | .02 | .792 | -.15 | .20 |  | **.17** | **.049** | **.001** | **.33** |  | .02 | .862 | -.16 | .19 |  | .997 | .066 |
| Shared Meat Consumption | Openness/Intellect | **-.11** | **.039** | **-.22** | **-.01** |  |  |  |  |  |  | -.09 | .115 | -.20 | .02 |  |  |  |  |  |  | .997 | .069 |
|  | Openness | **-.13** | **.020** | **-.24** | **-.02** |  |  |  |  |  |  | -.02 | .775 | -.13 | .10 |  |  |  |  |  |  | .995 | .062 |
|  | Intellect | -.06 | .247 | -.17 | .04 |  |  |  |  |  |  | -.11 | .040 | -.22 | -.01 |  |  |  |  |  |  | .996 | .078 |
|  | Agreeableness | -.11 | .057 | -.22 | .003 |  |  |  |  |  |  | -.01 | .917 | -.12 | .10 |  |  |  |  |  |  | .998 | .070 |
|  | Politeness | -.04 | .510 | -.15 | .07 |  |  |  |  |  |  | .03 | .612 | -.08 | .14 |  |  |  |  |  |  | .996 | .071 |
|  | Compassion | **-.15** | **.009** | **-.26** | **-.04** |  |  |  |  |  |  | -.04 | .490 | -.15 | .07 |  |  |  |  |  |  | .998 | .066 |
|  | Conscientiousness | -.002 | .971 | -.11 | .11 |  |  |  |  |  |  | .05 | .423 | -.07 | .16 |  |  |  |  |  |  | .996 | .090 |
|  | Industriousness | -.02 | .777 | -.13 | .10 |  |  |  |  |  |  | .03 | .650 | -.09 | .14 |  |  |  |  |  |  | .994 | .102 |
|  | Orderliness | .02 | .724 | -.09 | .13 |  |  |  |  |  |  | .05 | .396 | -.06 | .16 |  |  |  |  |  |  | .995 | .070 |
|  | Neuroticism | .05 | .388 | -.06 | .16 |  |  |  |  |  |  | -.10 | .075 | -.22 | .01 |  |  |  |  |  |  | .992 | .097 |
|  | Withdrawal | .02 | .737 | -.09 | .13 |  |  |  |  |  |  | **-.13** | **.021** | **-.25** | **-.02** |  |  |  |  |  |  | .991 | .109 |
|  | Volatility | .07 | .215 | -.04 | .18 |  |  |  |  |  |  | -.05 | .387 | -.16 | .06 |  |  |  |  |  |  | .992 | .077 |
|  | Extraversion | -.10 | .063 | -.21 | .01 |  |  |  |  |  |  | .06 | .278 | -.05 | .17 |  |  |  |  |  |  | .997 | .073 |
|  | Assertiveness | -.09 | .099 | -.20 | .02 |  |  |  |  |  |  | .07 | .212 | -.04 | .18 |  |  |  |  |  |  | .996 | .076 |
|  | Enthusiasm | -.09 | .116 | -.20 | .02 |  |  |  |  |  |  | .04 | .516 | -.07 | .15 |  |  |  |  |  |  | .997 | .069 |
| Self-Report | Openness/Intellect | **-.29** | **< .001** | **-.45** | **-.14** |  | -.12 | .161 | -.29 | .05 |  | -.09 | .286 | -.25 | .07 |  | -.08 | .323 | -.25 | .08 |  | .998 | .064 |
|  | Openness | **-.20** | **.013** | **-.37** | **-.04** |  | -.12 | .159 | -.29 | .05 |  | -.10 | .238 | -.26 | .06 |  | .04 | .610 | -.13 | .21 |  | .996 | .062 |
|  | Intellect | **-.26** | **.001** | **-.42** | **-.10** |  | -.10 | .238 | -.26 | .06 |  | -.07 | .420 | -.23 | .10 |  | **-.21** | **.011** | **-.36** | **-.05** |  | .997 | .072 |
|  | Agreeableness | -.16 | .058 | -.32 | .01 |  | -.07 | .422 | -.24 | .10 |  | .02 | .820 | -.15 | .18 |  | -.01 | .952 | -.17 | .16 |  | .998 | .063 |
|  | Politeness | -.09 | .284 | -.26 | .08 |  | .03 | .721 | -.14 | .20 |  | .09 | .297 | -.08 | .26 |  | .04 | .629 | -.13 | .21 |  | .997 | .066 |
|  | Compassion | **-.20** | **.020** | **-.36** | **-.03** |  | -.15 | .088 | -.31 | .02 |  | -.06 | .459 | -.23 | .10 |  | -.08 | .367 | -.25 | .09 |  | .998 | .058 |
|  | Conscientiousness | .08 | .360 | -.09 | .24 |  | -.09 | .293 | -.26 | .08 |  | .03 | .686 | -.13 | .20 |  | .07 | .421 | -.10 | .23 |  | .996 | .082 |
|  | Industriousness | .05 | .562 | -.12 | .22 |  | -.08 | .344 | -.25 | .09 |  | -.01 | .898 | -.18 | .16 |  | .03 | .722 | -.14 | .20 |  | .994 | .093 |
|  | Orderliness | .08 | .333 | -.08 | .25 |  | -.07 | .407 | -.24 | .10 |  | .06 | .444 | -.10 | .23 |  | .09 | .265 | -.07 | .26 |  | .996 | .062 |
|  | Neuroticism | .05 | .534 | -.11 | .22 |  | -.10 | .257 | -.27 | .07 |  | -.004 | .962 | -.17 | .17 |  | .11 | .179 | -.05 | .28 |  | .993 | .090 |
|  | Withdrawal | -.06 | .456 | -.23 | .10 |  | -.11 | .214 | -.29 | .06 |  | .02 | .799 | -.15 | .19 |  | .03 | .740 | -.14 | .20 |  | .992 | .097 |
|  | Volatility | .15 | .064 | -.01 | .32 |  | -.08 | .371 | -.24 | .09 |  | -.03 | .693 | -.20 | .13 |  | **.17** | **.035** | **.01** | **.34** |  | .993 | .075 |
|  | Extraversion | -.02 | .844 | -.18 | .15 |  | .02 | .776 | -.14 | .19 |  | -.01 | .939 | -.17 | .16 |  | -.01 | .889 | -.18 | .16 |  | .997 | .064 |
|  | Assertiveness | -.02 | .828 | -.19 | .15 |  | .03 | .739 | -.14 | .20 |  | -.03 | .713 | -.20 | .14 |  | .002 | .984 | -.17 | .17 |  | .996 | .068 |
|  | Enthusiasm | -.01 | .891 | -.18 | .16 |  | .01 | .871 | -.15 | .18 |  | .02 | .824 | -.15 | .18 |  | -.02 | .805 | -.19 | .15 |  | .997 | .064 |
| Informant-Report | Openness/Intellect | **-.18** | **.028** | **-.34** | **-.02** |  | -.05 | .563 | -.21 | .12 |  | -.13 | .113 | -.29 | .03 |  | -.13 | .123 | -.29 | .03 |  | .998 | .060 |
|  | Openness | **-.17** | **.034** | **-.33** | **-.01** |  | -.02 | .788 | -.19 | .14 |  | -.14 | .082 | -.30 | .02 |  | -.12 | .150 | -.29 | .04 |  | .996 | .061 |
|  | Intellect | -.11 | .203 | -.27 | .06 |  | -.05 | .566 | -.22 | .12 |  | -.05 | .549 | -.22 | .12 |  | -.08 | .319 | -.25 | .08 |  | .996 | .071 |
|  | Agreeableness | -.05 | .580 | -.22 | .12 |  | -.08 | .329 | -.25 | .08 |  | -.10 | .250 | -.26 | .07 |  | -.01 | .945 | -.17 | .16 |  | .997 | .067 |
|  | Politeness | .01 | .890 | -.16 | .18 |  | -.11 | .212 | -.27 | .06 |  | -.11 | .185 | -.28 | .05 |  | .06 | .445 | -.10 | .23 |  | .996 | .069 |
|  | Compassion | -.09 | .315 | -.26 | .08 |  | -.04 | .635 | -.21 | .13 |  | -.05 | .515 | -.22 | .11 |  | -.07 | .422 | -.24 | .10 |  | .997 | .061 |
|  | Conscientiousness | -.05 | .538 | -.22 | .12 |  | .07 | .429 | -.10 | .24 |  | .02 | .795 | -.15 | .19 |  | -.05 | .599 | -.22 | .13 |  | .996 | .079 |
|  | Industriousness | -.05 | .595 | -.22 | .12 |  | .04 | .613 | -.12 | .21 |  | .06 | .475 | -.11 | .23 |  | -.03 | .693 | -.21 | .14 |  | .995 | .086 |
|  | Orderliness | -.05 | .561 | -.22 | .12 |  | .08 | .372 | -.09 | .25 |  | -.01 | .877 | -.19 | .16 |  | -.04 | .645 | -.21 | .13 |  | .995 | .069 |
|  | Neuroticism | -.01 | .891 | -.19 | .16 |  | .04 | .674 | -.13 | .20 |  | -.11 | .203 | -.28 | .06 |  | **-.21** | **.016** | **-.38** | **-.04** |  | .993 | .078 |
|  | Withdrawal | -.03 | .760 | -.20 | .14 |  | -.02 | .851 | -.18 | .15 |  | **-.20** | **.018** | **-.36** | **-.03** |  | **-.19** | **.032** | **-.36** | **-.02** |  | .993 | .089 |
|  | Volatility | .02 | .855 | -.16 | .19 |  | .09 | .271 | -.07 | .26 |  | < .001 | .997 | -.17 | .17 |  | **-.19** | **.023** | **-.36** | **-.03** |  | .992 | .068 |
|  | Extraversion | -.11 | .188 | -.28 | .06 |  | -.02 | .833 | -.19 | .15 |  | .08 | .346 | -.09 | .25 |  | .06 | .503 | -.11 | .23 |  | .998 | .066 |
|  | Assertiveness | -.12 | .171 | -.29 | .05 |  | .05 | .533 | -.12 | .23 |  | .09 | .284 | -.08 | .26 |  | .05 | .582 | -.12 | .22 |  | .997 | .070 |
|  | Enthusiasm | -.07 | .409 | -.24 | .10 |  | -.07 | .385 | -.24 | .09 |  | .05 | .567 | -.12 | .22 |  | .06 | .517 | -.11 | .23 |  | .997 | .060 |
| Only Meat Consumption | Openness/Intellect | **-.31** | **< .001** | **-.48** | **-.14** |  | -.02 | .868 | -.20 | .17 |  | -.03 | .742 | -.20 | .15 |  | -.15 | .115 | -.33 | .04 |  | .998 | .064 |
|  | Openness | **-.28** | **.004** | **-.46** | **-.09** |  | -.06 | .570 | -.26 | .14 |  | -.01 | .924 | -.20 | .18 |  | -.02 | .844 | -.23 | .18 |  | .997 | .055 |
|  | Intellect | **-.23** | **.008** | **-.40** | **-.06** |  | .01 | .951 | -.17 | .18 |  | -.03 | .774 | -.20 | .15 |  | **-.21** | **.018** | **-.38** | **-.03** |  | .997 | .077 |
|  | Agreeableness | -.12 | .175 | -.29 | .05 |  | -.06 | .508 | -.23 | .12 |  | -.005 | .957 | -.18 | .17 |  | .06 | .535 | -.12 | .23 |  | .998 | .067 |
|  | Politeness | -.02 | .818 | -.20 | .16 |  | -.08 | .408 | -.26 | .11 |  | -.03 | .788 | -.21 | .16 |  | .13 | .170 | -.05 | .31 |  | .997 | .067 |
|  | Compassion | **-.17** | **.048** | **-.34** | **-.001** |  | -.04 | .607 | -.22 | .13 |  | .01 | .910 | -.16 | .18 |  | -.02 | .857 | -.19 | .16 |  | .998 | .062 |
|  | Conscientiousness | -.02 | .813 | -.22 | .17 |  | -.04 | .718 | -.23 | .16 |  | .03 | .735 | -.16 | .23 |  | -.003 | .975 | -.20 | .20 |  | .997 | .086 |
|  | Industriousness | -.03 | .797 | -.22 | .17 |  | -.02 | .806 | -.21 | .17 |  | .02 | .818 | -.17 | .21 |  | -.04 | .666 | -.23 | .15 |  | .995 | .100 |
|  | Orderliness | -.01 | .945 | -.19 | .18 |  | -.04 | .710 | -.22 | .15 |  | .03 | .765 | -.16 | .21 |  | .03 | .726 | -.15 | .22 |  | .996 | .066 |
|  | Neuroticism | .10 | .311 | -.09 | .28 |  | .03 | .728 | -.15 | .22 |  | -.08 | .376 | -.27 | .10 |  | -.07 | .459 | -.26 | .12 |  | .994 | .092 |
|  | Withdrawal | .002 | .980 | -.19 | .19 |  | -.01 | .924 | -.20 | .18 |  | -.10 | .269 | -.29 | .08 |  | -.08 | .426 | -.28 | .12 |  | .993 | .102 |
|  | Volatility | .16 | .085 | -.02 | .34 |  | .07 | .427 | -.11 | .26 |  | -.03 | .716 | -.21 | .15 |  | -.05 | .595 | -.24 | .14 |  | .993 | .074 |
|  | Extraversion | -.15 | .080 | -.33 | .02 |  | .02 | .833 | -.16 | .20 |  | .15 | .078 | -.02 | .32 |  | .02 | .823 | -.16 | .20 |  | .997 | .069 |
|  | Assertiveness | **-.20** | **.024** | **-.37** | **-.03** |  | .05 | .550 | -.12 | .23 |  | .16 | .072 | -.01 | .33 |  | -.03 | .781 | -.21 | .15 |  | .997 | .071 |
|  | Enthusiasm | -.08 | .382 | -.25 | .10 |  | -.01 | .880 | -.19 | .16 |  | .11 | .204 | -.06 | .28 |  | .05 | .562 | -.12 | .23 |  | .997 | .065 |
| Only Fish Consumption | Openness/Intellect | -.08 | .164 | -.19 | .03 |  |  |  |  |  |  | .004 | .944 | -.11 | .12 |  |  |  |  |  |  | .997 | .064 |
|  | Openness | .02 | .794 | -.10 | .13 |  |  |  |  |  |  | -.01 | .917 | -.12 | .11 |  |  |  |  |  |  | .996 | .057 |
|  | Intellect | **-.15** | **.008** | **-.25** | **-.04** |  |  |  |  |  |  | -.01 | .841 | -.12 | .10 |  |  |  |  |  |  | .997 | .076 |
|  | Agreeableness | -.06 | .253 | -.17 | .05 |  |  |  |  |  |  | -.05 | .342 | -.16 | .06 |  |  |  |  |  |  | .998 | .066 |
|  | Politeness | -.01 | .854 | -.12 | .10 |  |  |  |  |  |  | -.03 | .608 | -.14 | .08 |  |  |  |  |  |  | .997 | .066 |
|  | Compassion | -.09 | .093 | -.20 | .02 |  |  |  |  |  |  | -.06 | .251 | -.17 | .05 |  |  |  |  |  |  | .998 | .063 |
|  | Conscientiousness | .05 | .360 | -.06 | .17 |  |  |  |  |  |  | .06 | .338 | -.06 | .17 |  |  |  |  |  |  | .996 | .087 |
|  | Industriousness | .02 | .729 | -.09 | .13 |  |  |  |  |  |  | .07 | .255 | -.05 | .18 |  |  |  |  |  |  | .995 | .100 |
|  | Orderliness | .07 | .198 | -.04 | .18 |  |  |  |  |  |  | .03 | .567 | -.08 | .14 |  |  |  |  |  |  | .996 | .067 |
|  | Neuroticism | .04 | .489 | -.07 | .15 |  |  |  |  |  |  | -.05 | .410 | -.16 | .07 |  |  |  |  |  |  | .993 | .095 |
|  | Withdrawal | -.01 | .800 | -.13 | .10 |  |  |  |  |  |  | -.001 | .987 | -.12 | .11 |  |  |  |  |  |  | .992 | .108 |
|  | Volatility | .08 | .137 | -.03 | .19 |  |  |  |  |  |  | -.08 | .153 | -.19 | .03 |  |  |  |  |  |  | .993 | .074 |
|  | Extraversion | -.03 | .592 | -.14 | .08 |  |  |  |  |  |  | .03 | .549 | -.08 | .15 |  |  |  |  |  |  | .997 | .071 |
|  | Assertiveness | .01 | .801 | -.10 | .12 |  |  |  |  |  |  | .06 | .306 | -.05 | .17 |  |  |  |  |  |  | .997 | .071 |
|  | Enthusiasm | -.06 | .262 | -.17 | .05 |  |  |  |  |  |  | .002 | .975 | -.11 | .11 |  |  |  |  |  |  | .997 | .065 |

*Note.* Bolded rows indicate statistically significant effects (i.e., *p* < .05); The Actor Partner Interdependence Model for Shared Meat Consumption, and Only Fish Consumption was indistinguishable and only one actor and partner effect for each trait is estimated; 95% Confidence intervals are reported; GFI = Goodness of Fit Index; SRMR = Standardized Root Mean Squared; Each model includes age, education, and income as covariates.

## Table S16

*Constrained Standardized Actor and Partner Effects for Big Five Domain and Aspect Traits Split by Model.*

| Model |  | Actor Effect | | | |  | Partner Effect | | | |  |  |
| --- | --- | --- | --- | --- | --- | --- | --- | --- | --- | --- | --- | --- |
|  | Trait | *ß* | *p* | Lower CI | Higher CI |  | *ß* | *p* | Lower CI | Higher CI | GFI | SRMR |
| Combined Meat Consumption | OI | **-.18** | **.002** | **-.29** | **-.07** |  | -.07 | .231 | -.18 | .04 | .998 | .063 |
|  | Openness | **-.16** | **.008** | **-.27** | **-.04** |  | -.02 | .700 | -.14 | .09 | .997 | .054 |
|  | Intellect | **-.14** | **.014** | **-.25** | **-.03** |  | -.08 | .152 | -.19 | .03 | .997 | .075 |
|  | Agreeableness | **-.13** | **.029** | **-.24** | **-.01** |  | .04 | .537 | -.08 | .15 | .998 | .066 |
|  | Politeness | -.07 | .249 | -.18 | .05 |  | .07 | .253 | -.05 | .18 | .997 | .066 |
|  | Compassion | **-.15** | **.008** | **-.26** | **-.04** |  | -.002 | .974 | -.11 | .11 | .998 | .061 |
|  | Conscientiousness | -.04 | .458 | -.16 | .07 |  | .06 | .348 | -.06 | .17 | .997 | .086 |
|  | Industriousness | -.05 | .415 | -.16 | .07 |  | .04 | .486 | -.08 | .16 | .995 | .100 |
|  | Orderliness | -.01 | .799 | -.13 | .10 |  | .04 | .466 | -.07 | .16 | .996 | .066 |
|  | Neuroticism | .07 | .204 | -.04 | .19 |  | **-.12** | **.047** | **-.23** | **-.002** | .994 | .091 |
|  | Withdrawal | .01 | .871 | -.11 | .13 |  | -.11 | .059 | -.23 | .004 | .993 | .102 |
|  | Volatility | **.12** | **.032** | **.01** | **.24** |  | -.10 | .103 | -.21 | .02 | .993 | .073 |
|  | Extraversion | -.11 | .052 | -.22 | .001 |  | .10 | .096 | -.02 | .21 | .998 | .068 |
|  | Assertiveness | -.09 | .108 | -.20 | .02 |  | .09 | .137 | -.03 | .20 | .997 | .070 |
|  | Enthusiasm | -.10 | .083 | -.21 | .01 |  | .08 | .170 | -.03 | .19 | .997 | .064 |
| General Meat Consumption | OI | **-.22** | **< .001** | **-.34** | **-.11** |  | -.03 | .609 | -.15 | .09 | .998 | .065 |
|  | Openness | **-.16** | **.013** | **-.28** | **-.03** |  | -.02 | .706 | -.15 | .10 | .997 | .056 |
|  | Intellect | **-.20** | **.001** | **-.32** | **-.09** |  | -.03 | .639 | -.14 | .09 | .998 | .077 |
|  | Agreeableness | **-.13** | **.030** | **-.24** | **-.01** |  | .08 | .200 | -.04 | .19 | .998 | .068 |
|  | Politeness | -.09 | .131 | -.21 | .03 |  | .10 | .115 | -.02 | .22 | .997 | .068 |
|  | Compassion | **-.14** | **.021** | **-.25** | **-.02** |  | .04 | .482 | -.07 | .16 | .998 | .063 |
|  | Conscientiousness | -.09 | .165 | -.21 | .04 |  | .07 | .310 | -.06 | .19 | .997 | .088 |
|  | Industriousness | -.08 | .203 | -.20 | .04 |  | .06 | .360 | -.07 | .18 | .996 | .101 |
|  | Orderliness | -.05 | .382 | -.17 | .07 |  | .03 | .580 | -.09 | .16 | .997 | .068 |
|  | Neuroticism | .09 | .129 | -.03 | .21 |  | -.12 | .055 | -.24 | .003 | .994 | .092 |
|  | Withdrawal | < .001 | .994 | -.12 | .12 |  | -.08 | .228 | -.20 | .05 | .994 | .103 |
|  | Volatility | **.16** | **.006** | **.05** | **.28** |  | **-.13** | **.028** | **-.25** | **-.01** | .994 | .075 |
|  | Extraversion | -.10 | .085 | -.22 | .01 |  | **.12** | **.037** | **.01** | **.24** | .998 | .070 |
|  | Assertiveness | -.08 | .202 | -.19 | .04 |  | .09 | .125 | -.03 | .21 | .997 | .071 |
|  | Enthusiasm | -.10 | .098 | -.21 | .02 |  | **.12** | **.045** | **.003** | **.23** | .997 | .066 |
| Shared Meat Consumption | OI | **-.11** | **.039** | **-.22** | **-.01** |  | -.09 | .115 | -.20 | .02 | .997 | .069 |
|  | Openness | **-.13** | **.020** | **-.24** | **-.02** |  | -.02 | .775 | -.13 | .10 | .995 | .062 |
|  | Intellect | -.06 | .247 | -.17 | .04 |  | **-.11** | **.040** | **-.22** | **-.01** | .996 | .078 |
|  | Agreeableness | -.11 | .057 | -.22 | .003 |  | -.01 | .917 | -.12 | .10 | .998 | .070 |
|  | Politeness | -.04 | .510 | -.15 | .07 |  | .03 | .612 | -.08 | .14 | .996 | .071 |
|  | Compassion | **-.15** | **.009** | **-.26** | **-.04** |  | -.04 | .490 | -.15 | .07 | .998 | .066 |
|  | Conscientiousness | -.002 | .971 | -.11 | .11 |  | .05 | .423 | -.07 | .16 | .996 | .090 |
|  | Industriousness | -.02 | .777 | -.13 | .10 |  | .03 | .650 | -.09 | .14 | .994 | .102 |
|  | Orderliness | .02 | .724 | -.09 | .13 |  | .05 | .396 | -.06 | .16 | .995 | .070 |
|  | Neuroticism | .05 | .388 | -.06 | .16 |  | -.10 | .075 | -.22 | .01 | .992 | .097 |
|  | Withdrawal | .02 | .737 | -.09 | .13 |  | **-.13** | **.021** | **-.25** | **-.02** | .991 | .109 |
|  | Volatility | .07 | .215 | -.04 | .18 |  | -.05 | .387 | -.16 | .06 | .992 | .077 |
|  | Extraversion | -.10 | .063 | -.21 | .01 |  | .06 | .278 | -.05 | .17 | .997 | .073 |
|  | Assertiveness | -.09 | .099 | -.20 | .02 |  | .07 | .212 | -.04 | .18 | .996 | .076 |
|  | Enthusiasm | -.09 | .116 | -.20 | .02 |  | .04 | .516 | -.07 | .15 | .997 | .069 |
| Self-Report | OI | **-.22** | **< .001** | **-.32** | **-.11** |  | -.08 | .181 | -.19 | .04 | .998 | .064 |
|  | Openness | **-.17** | **.002** | **-.28** | **-.06** |  | -.02 | .792 | -.13 | .10 | .996 | .062 |
|  | Intellect | **-.19** | **.001** | **-.30** | **-.08** |  | **-.14** | **.018** | **-.25** | **-.02** | .997 | .072 |
|  | Agreeableness | -.12 | .063 | -.24 | .01 |  | .03 | .657 | -.10 | .15 | .998 | .063 |
|  | Politeness | -.03 | .612 | -.14 | .08 |  | .09 | .144 | -.03 | .20 | .997 | .066 |
|  | Compassion | **-.18** | **.008** | **-.31** | **-.05** |  | -.06 | .396 | -.19 | .08 | .998 | .058 |
|  | Conscientiousness | -.02 | .723 | -.14 | .10 |  | .04 | .476 | -.08 | .17 | .996 | .082 |
|  | Industriousness | -.03 | .627 | -.15 | .09 |  | -.001 | .990 | -.12 | .12 | .994 | .093 |
|  | Orderliness | -.01 | .873 | -.13 | .11 |  | .08 | .196 | -.04 | .20 | .996 | .062 |
|  | Neuroticism | -.03 | .631 | -.16 | .10 |  | .07 | .259 | -.06 | .20 | .993 | .090 |
|  | Withdrawal | -.09 | .176 | -.22 | .04 |  | .05 | .461 | -.08 | .18 | .992 | .097 |
|  | Volatility | .03 | .670 | -.10 | .15 |  | .09 | .176 | -.04 | .21 | .993 | .075 |
|  | Extraversion | -.03 | .675 | -.15 | .09 |  | -.01 | .846 | -.13 | .11 | .997 | .064 |
|  | Assertiveness | -.02 | .743 | -.14 | .10 |  | -.02 | .726 | -.15 | .10 | .996 | .068 |
|  | Enthusiasm | -.02 | .692 | -.14 | .09 |  | .001 | .989 | -.12 | .12 | .997 | .064 |
| Informant-Report | OI | -.11 | .067 | -.23 | .01 |  | **-.12** | **.038** | **-.24** | **-.01** | .998 | .060 |
|  | Openness | -.09 | .120 | -.21 | .02 |  | **-.13** | **.030** | **-.25** | **-.01** | .996 | .061 |
|  | Intellect | -.07 | .215 | -.19 | .04 |  | -.06 | .316 | -.18 | .06 | .996 | .071 |
|  | Agreeableness | -.07 | .231 | -.19 | .05 |  | -.02 | .716 | -.14 | .10 | .997 | .067 |
|  | Politeness | -.05 | .387 | -.17 | .07 |  | -.003 | .965 | -.12 | .11 | .996 | .069 |
|  | Compassion | -.07 | .253 | -.19 | .05 |  | -.04 | .560 | -.16 | .09 | .997 | .061 |
|  | Conscientiousness | -.001 | .988 | -.13 | .13 |  | 3e-04 | .996 | -.13 | .13 | .996 | .079 |
|  | Industriousness | -.01 | .843 | -.14 | .11 |  | .03 | .657 | -.10 | .15 | .995 | .086 |
|  | Orderliness | .01 | .932 | -.13 | .14 |  | -.02 | .740 | -.16 | .11 | .995 | .069 |
|  | Neuroticism | .03 | .666 | -.11 | .17 |  | **-.18** | **.009** | **-.32** | **-.05** | .993 | .078 |
|  | Withdrawal | -.004 | .951 | -.14 | .13 |  | **-.21** | **.002** | **-.34** | **-.08** | .993 | .089 |
|  | Volatility | .07 | .270 | -.06 | .21 |  | -.11 | .086 | -.24 | .02 | .992 | .068 |
|  | Extraversion | -.08 | .222 | -.21 | .05 |  | .08 | .209 | -.05 | .21 | .998 | .066 |
|  | Assertiveness | -.05 | .428 | -.19 | .08 |  | .09 | .208 | -.05 | .22 | .997 | .070 |
|  | Enthusiasm | -.08 | .210 | -.21 | .05 |  | .06 | .360 | -.07 | .19 | .997 | .060 |
| Only Meat Consumption | OI | **-.17** | **.003** | **-.28** | **-.06** |  | -.08 | .182 | -.19 | .04 | .998 | .064 |
|  | Openness | **-.17** | **.004** | **-.29** | **-.05** |  | -.01 | .875 | -.13 | .11 | .997 | .055 |
|  | Intellect | **-.12** | **.039** | **-.23** | **-.01** |  | -.10 | .067 | -.22 | .01 | .997 | .077 |
|  | Agreeableness | -.10 | .073 | -.22 | .01 |  | .06 | .309 | -.05 | .17 | .998 | .067 |
|  | Politeness | -.06 | .324 | -.17 | .06 |  | .08 | .190 | -.04 | .19 | .997 | .067 |
|  | Compassion | **-.13** | **.030** | **-.24** | **-.01** |  | .03 | .642 | -.09 | .14 | .998 | .062 |
|  | Conscientiousness | -.07 | .263 | -.18 | .05 |  | .04 | .512 | -.08 | .16 | .997 | .086 |
|  | Industriousness | -.05 | .381 | -.17 | .06 |  | .01 | .912 | -.11 | .13 | .995 | .100 |
|  | Orderliness | -.05 | .397 | -.16 | .06 |  | .05 | .428 | -.07 | .16 | .996 | .066 |
|  | Neuroticism | .08 | .176 | -.04 | .20 |  | -.08 | .171 | -.20 | .04 | .994 | .092 |
|  | Withdrawal | .01 | .823 | -.10 | .13 |  | -.09 | .119 | -.21 | .02 | .993 | .102 |
|  | Volatility | **.13** | **.025** | **.02** | **.24** |  | -.05 | .389 | -.17 | .06 | .993 | .074 |
|  | Extraversion | -.11 | .061 | -.22 | .005 |  | .11 | .060 | -.005 | .22 | .997 | .069 |
|  | Assertiveness | -.11 | .045 | -.23 | -.003 |  | .09 | .112 | -.02 | .21 | .997 | .071 |
|  | Enthusiasm | -.07 | .206 | -.19 | .04 |  | .10 | .098 | -.02 | .21 | .997 | .065 |
| Only Fish Consumption | OI | -.08 | .164 | -.19 | .03 |  | .004 | .944 | -.11 | .12 | .997 | .064 |
|  | Openness | .02 | .794 | -.10 | .13 |  | -.01 | .917 | -.12 | .11 | .996 | .057 |
|  | Intellect | **-.15** | **.008** | **-.25** | **-.04** |  | -.01 | .841 | -.12 | .10 | .997 | .076 |
|  | Agreeableness | -.06 | .253 | -.17 | .05 |  | -.05 | .342 | -.16 | .06 | .998 | .066 |
|  | Politeness | -.01 | .854 | -.12 | .10 |  | -.03 | .608 | -.14 | .08 | .997 | .066 |
|  | Compassion | -.09 | .093 | -.20 | .02 |  | -.06 | .251 | -.17 | .05 | .998 | .063 |
|  | Conscientiousness | .05 | .360 | -.06 | .17 |  | .06 | .338 | -.06 | .17 | .996 | .087 |
|  | Industriousness | .02 | .729 | -.09 | .13 |  | .07 | .255 | -.05 | .18 | .995 | .100 |
|  | Orderliness | .07 | .198 | -.04 | .18 |  | .03 | .567 | -.08 | .14 | .996 | .067 |
|  | Neuroticism | .04 | .489 | -.07 | .15 |  | -.05 | .410 | -.16 | .07 | .993 | .095 |
|  | Withdrawal | -.01 | .800 | -.13 | .10 |  | -.001 | .987 | -.12 | .11 | .992 | .108 |
|  | Volatility | .08 | .137 | -.03 | .19 |  | -.08 | .153 | -.19 | .03 | .993 | .074 |
|  | Extraversion | -.03 | .592 | -.14 | .08 |  | .03 | .549 | -.08 | .15 | .997 | .071 |
|  | Assertiveness | .01 | .801 | -.10 | .12 |  | .06 | .306 | -.05 | .17 | .997 | .071 |
|  | Enthusiasm | -.06 | .262 | -.17 | .05 |  | .002 | .975 | -.11 | .11 | .997 | .065 |

*Note.* Bolded rows indicate statistically significant effects (i.e., *p* < .05); 95% Confidence intervals are reported; GFI = Goodness of Fit Index; SRMR = Standardized Root Mean Squared; Each model includes age, education, income, and gender as covariates.

## Table S17

*Standardized Actor and Partner Effects for Big Five Domain and Aspect Traits Split by Model Whilst Controlling for Gender.*

|  |  | Actor Effect (Higher Meat Consumer) | | | |  | Actor Effect (Lower Meat Consumer) | | | |  | Partner Effect (Higher Meat Consumer) | | | |  | Partner Effect (Lower Meat Consumer) | | | |  |  |  |
| --- | --- | --- | --- | --- | --- | --- | --- | --- | --- | --- | --- | --- | --- | --- | --- | --- | --- | --- | --- | --- | --- | --- | --- |
| Model | Trait | *ß* | *p* | Lower CI | Higher CI |  | *ß* | *p* | Lower CI | Higher CI |  | *ß* | *p* | Lower CI | Higher CI |  | *ß* | *p* | Lower CI | Higher CI |  | GFI | SRMR |
| Combined Meat Consumption | Openness/Intellect | **-.30** | **.001** | **-.47** | **-.13** |  | -.11 | .123 | -.26 | .03 |  | -.02 | .801 | -.20 | .15 |  | -.12 | .187 | -.31 | .06 |  | .993 | .110 |
|  | Openness | **-.23** | **.021** | **-.42** | **-.03** |  | -.08 | .266 | -.23 | .06 |  | -.04 | .687 | -.23 | .15 |  | -.03 | .798 | -.23 | .18 |  | .989 | .108 |
|  | Intellect | **-.15** | **.010** | **-.26** | **-.04** |  |  |  |  |  |  | -.07 | .248 | -.18 | .05 |  |  |  |  |  |  | .990 | .125 |
|  | Agreeableness | **-.12** | **.043** | **-.23** | **-.004** |  |  |  |  |  |  | .01 | .807 | -.10 | .13 |  |  |  |  |  |  | .994 | .121 |
|  | Politeness | -.07 | .239 | -.18 | .04 |  |  |  |  |  |  | .05 | .429 | -.07 | .16 |  |  |  |  |  |  | .990 | .121 |
|  | Compassion | **-.13** | **.020** | **-.25** | **-.02** |  |  |  |  |  |  | -.02 | .765 | -.13 | .10 |  |  |  |  |  |  | .992 | .121 |
|  | Conscientiousness | -.02 | .693 | -.14 | .09 |  |  |  |  |  |  | .05 | .364 | -.06 | .17 |  |  |  |  |  |  | .989 | .129 |
|  | Industriousness | -.03 | .622 | -.14 | .09 |  |  |  |  |  |  | .04 | .449 | -.07 | .16 |  |  |  |  |  |  | .986 | .135 |
|  | Orderliness | -.001 | .989 | -.11 | .11 |  |  |  |  |  |  | .04 | .506 | -.07 | .15 |  |  |  |  |  |  | .987 | .120 |
|  | Neuroticism | .06 | .343 | -.06 | .17 |  |  |  |  |  |  | -.09 | .138 | -.20 | .03 |  |  |  |  |  |  | .979 | .136 |
|  | Withdrawal | -.01 | .833 | -.13 | .10 |  |  |  |  |  |  | -.10 | .092 | -.21 | .02 |  |  |  |  |  |  | .978 | .140 |
|  | Volatility | .11 | .053 | -.001 | .23 |  |  |  |  |  |  | -.06 | .351 | -.17 | .06 |  |  |  |  |  |  | .976 | .131 |
|  | Extraversion | -.08 | .150 | -.20 | .03 |  |  |  |  |  |  | .08 | .179 | -.04 | .19 |  |  |  |  |  |  | .991 | .125 |
|  | Assertiveness | -.06 | .328 | -.17 | .06 |  |  |  |  |  |  | .06 | .269 | -.05 | .18 |  |  |  |  |  |  | .988 | .127 |
|  | Enthusiasm | -.08 | .152 | -.20 | .03 |  |  |  |  |  |  | .07 | .255 | -.05 | .18 |  |  |  |  |  |  | .989 | .121 |
| General Meat Consumption | Openness/Intellect | **-.35** | **< .001** | **-.52** | **-.19** |  | -.15 | .055 | -.29 | .003 |  | .03 | .697 | -.14 | .21 |  | -.14 | .139 | -.32 | .04 |  | .994 | .111 |
|  | Openness | **-.22** | **.028** | **-.41** | **-.02** |  | -.12 | .133 | -.27 | .04 |  | -.05 | .616 | -.24 | .14 |  | -.03 | .773 | -.23 | .17 |  | .990 | .108 |
|  | Intellect | **-.34** | **< .001** | **-.49** | **-.18** |  | **-.18** | **.017** | **-.33** | **-.03** |  | .10 | .235 | -.06 | .26 |  | **-.19** | **.029** | **-.36** | **-.02** |  | .993 | .117 |
|  | Agreeableness | -.16 | .066 | -.33 | .01 |  | -.12 | .133 | -.28 | .04 |  | .05 | .545 | -.12 | .23 |  | -.02 | .787 | -.20 | .15 |  | .995 | .113 |
|  | Politeness | -.11 | .236 | -.29 | .07 |  | -.13 | .098 | -.29 | .02 |  | .07 | .460 | -.11 | .25 |  | .03 | .739 | -.15 | .22 |  | .992 | .113 |
|  | Compassion | -.17 | .051 | -.34 | .00 |  | -.13 | .120 | -.29 | .03 |  | .03 | .743 | -.14 | .20 |  | -.06 | .455 | -.23 | .11 |  | .994 | .113 |
|  | Conscientiousness | -.09 | .354 | -.28 | .10 |  | -.15 | .069 | -.30 | .01 |  | .10 | .295 | -.09 | .30 |  | -.07 | .495 | -.26 | .13 |  | .991 | .122 |
|  | Industriousness | -.09 | .365 | -.28 | .10 |  | -.15 | .067 | -.30 | .01 |  | .10 | .277 | -.08 | .29 |  | -.05 | .629 | -.24 | .14 |  | .989 | .129 |
|  | Orderliness | -.04 | .637 | -.22 | .14 |  | -.14 | .071 | -.30 | .01 |  | .05 | .593 | -.13 | .23 |  | -.06 | .553 | -.24 | .13 |  | .990 | .112 |
|  | Neuroticism | .11 | .265 | -.08 | .30 |  | -.12 | .153 | -.28 | .04 |  | -.14 | .122 | -.33 | .04 |  | -.04 | .713 | -.23 | .16 |  | .984 | .129 |
|  | Withdrawal | -.01 | .937 | -.20 | .18 |  | -.14 | .078 | -.30 | .02 |  | -.07 | .436 | -.26 | .11 |  | -.04 | .653 | -.24 | .15 |  | .984 | .131 |
|  | Volatility | **.19** | **.040** | **.01** | **.38** |  | -.09 | .254 | -.26 | .07 |  | **-.18** | **.050** | **-.35** | **-.00** |  | -.02 | .818 | -.22 | .17 |  | .982 | .124 |
|  | Extraversion | -.12 | .182 | -.30 | .06 |  | -.11 | .201 | -.27 | .06 |  | .14 | .124 | -.04 | .31 |  | .003 | .974 | -.17 | .18 |  | .992 | .117 |
|  | Assertiveness | -.08 | .360 | -.26 | .10 |  | -.13 | .135 | -.29 | .04 |  | .07 | .450 | -.11 | .25 |  | -.01 | .914 | -.19 | .17 |  | .990 | .118 |
|  | Enthusiasm | -.11 | .200 | -.28 | .06 |  | -.12 | .153 | -.28 | .04 |  | .15 | .082 | -.02 | .32 |  | .01 | .906 | -.16 | .18 |  | .991 | .113 |
| Shared Meat Consumption | Openness/Intellect | **-.12** | **.035** | **-.22** | **-.01** |  |  |  |  |  |  | -.08 | .141 | -.19 | .03 |  |  |  |  |  |  | .992 | .113 |
|  | Openness | **-.13** | **.023** | **-.24** | **-.02** |  |  |  |  |  |  | -.02 | .703 | -.13 | .09 |  |  |  |  |  |  | .986 | .111 |
|  | Intellect | -.07 | .216 | -.18 | .04 |  |  |  |  |  |  | -.10 | .072 | -.21 | .01 |  |  |  |  |  |  | .991 | .117 |
|  | Agreeableness | -.10 | .079 | -.21 | .01 |  |  |  |  |  |  | -.03 | .642 | -.14 | .08 |  |  |  |  |  |  | .994 | .114 |
|  | Politeness | -.04 | .491 | -.15 | .07 |  |  |  |  |  |  | .01 | .882 | -.10 | .12 |  |  |  |  |  |  | .991 | .115 |
|  | Compassion | **-.13** | **.021** | **-.24** | **-.02** |  |  |  |  |  |  | -.05 | .354 | -.16 | .06 |  |  |  |  |  |  | .993 | .114 |
|  | Conscientiousness | .02 | .753 | -.09 | .13 |  |  |  |  |  |  | .05 | .426 | -.07 | .16 |  |  |  |  |  |  | .990 | .123 |
|  | Industriousness | .002 | .967 | -.11 | .11 |  |  |  |  |  |  | .03 | .591 | -.08 | .14 |  |  |  |  |  |  | .987 | .130 |
|  | Orderliness | .03 | .551 | -.08 | .14 |  |  |  |  |  |  | .05 | .420 | -.06 | .16 |  |  |  |  |  |  | .988 | .113 |
|  | Neuroticism | .03 | .550 | -.08 | .15 |  |  |  |  |  |  | -.08 | .179 | -.19 | .04 |  |  |  |  |  |  | .981 | .131 |
|  | Withdrawal | .001 | .979 | -.11 | .11 |  |  |  |  |  |  | **-.12** | **.029** | **-.24** | **-.01** |  |  |  |  |  |  | .981 | .135 |
|  | Volatility | .06 | .291 | -.05 | .17 |  |  |  |  |  |  | -.01 | .843 | -.13 | .10 |  |  |  |  |  |  | .978 | .125 |
|  | Extraversion | -.08 | .163 | -.19 | .03 |  |  |  |  |  |  | .05 | .409 | -.06 | .16 |  |  |  |  |  |  | .992 | .118 |
|  | Assertiveness | -.06 | .277 | -.17 | .05 |  |  |  |  |  |  | .05 | .337 | -.06 | .17 |  |  |  |  |  |  | .989 | .120 |
|  | Enthusiasm | -.07 | .195 | -.18 | .04 |  |  |  |  |  |  | .03 | .652 | -.09 | .14 |  |  |  |  |  |  | .990 | .114 |
| Self-Report | Openness/Intellect | **-.29** | **< .001** | **-.44** | **-.13** |  | -.11 | .130 | -.26 | .03 |  | -.10 | .241 | -.25 | .06 |  | -.08 | .328 | -.25 | .08 |  | .992 | .111 |
|  | Openness | -.18 | .034 | -.35 | -.01 |  | -.08 | .305 | -.24 | .07 |  | -.12 | .168 | -.28 | .05 |  | .03 | .759 | -.15 | .20 |  | .985 | .118 |
|  | Intellect | **-.21** | **< .001** | **-.32** | **-.09** |  |  |  |  |  |  | -.11 | .071 | -.22 | .01 |  |  |  |  |  |  | .988 | .129 |
|  | Agreeableness | -.13 | .122 | -.30 | .04 |  | -.08 | .344 | -.24 | .09 |  | -.01 | .933 | -.18 | .17 |  | -.03 | .753 | -.20 | .15 |  | .993 | .126 |
|  | Politeness | -.07 | .409 | -.24 | .10 |  | -.10 | .194 | -.25 | .05 |  | .07 | .394 | -.10 | .25 |  | .03 | .750 | -.15 | .20 |  | .991 | .114 |
|  | Compassion | -.17 | .052 | -.34 | .001 |  | -.10 | .254 | -.26 | .07 |  | -.10 | .284 | -.27 | .08 |  | -.10 | .280 | -.27 | .08 |  | .991 | .131 |
|  | Conscientiousness | .08 | .317 | -.08 | .25 |  | -.12 | .119 | -.27 | .03 |  | .03 | .702 | -.13 | .20 |  | .07 | .412 | -.10 | .23 |  | .989 | .119 |
|  | Industriousness | -.01 | .836 | -.14 | .11 |  |  |  |  |  |  | .02 | .773 | -.10 | .14 |  |  |  |  |  |  | .983 | .132 |
|  | Orderliness | .09 | .258 | -.07 | .26 |  | -.12 | .122 | -.27 | .03 |  | .05 | .514 | -.11 | .22 |  | .09 | .302 | -.08 | .25 |  | .987 | .111 |
|  | Neuroticism | .09 | .282 | -.08 | .26 |  | -.15 | .070 | -.31 | .01 |  | -.05 | .556 | -.23 | .12 |  | .10 | .244 | -.07 | .27 |  | .978 | .138 |
|  | Withdrawal | -.03 | .744 | -.21 | .15 |  | -.10 | .215 | -.27 | .06 |  | -.002 | .978 | -.18 | .17 |  | .003 | .974 | -.17 | .18 |  | .976 | .141 |
|  | Volatility | **.18** | **.026** | **.02** | **.34** |  | -.17 | .029 | -.33 | -.02 |  | -.10 | .254 | -.27 | .07 |  | **.17** | **.044** | **.004** | **.33** |  | .978 | .130 |
|  | Extraversion | -.01 | .918 | -.13 | .11 |  |  |  |  |  |  | -.02 | .783 | -.14 | .10 |  |  |  |  |  |  | .989 | .120 |
|  | Assertiveness | -.01 | .929 | -.13 | .12 |  |  |  |  |  |  | -.01 | .861 | -.13 | .11 |  |  |  |  |  |  | .986 | .122 |
|  | Enthusiasm | -.005 | .935 | -.12 | .11 |  |  |  |  |  |  | -.02 | .767 | -.14 | .10 |  |  |  |  |  |  | .987 | .122 |
| Informant-Report | Openness/Intellect | **-.18** | **.024** | **-.34** | **-.02** |  | -.11 | .146 | -.26 | .04 |  | -.12 | .152 | -.28 | .04 |  | -.12 | .142 | -.29 | .04 |  | .992 | .110 |
|  | Openness | **-.18** | **.023** | **-.34** | **-.03** |  | -.11 | .171 | -.26 | .05 |  | -.12 | .151 | -.28 | .04 |  | -.11 | .213 | -.27 | .06 |  | .987 | .116 |
|  | Intellect | -.07 | .261 | -.19 | .05 |  |  |  |  |  |  | -.07 | .254 | -.19 | .05 |  |  |  |  |  |  | .987 | .122 |
|  | Agreeableness | -.07 | .446 | -.23 | .10 |  | -.12 | .136 | -.27 | .04 |  | -.09 | .300 | -.25 | .08 |  | .01 | .920 | -.16 | .18 |  | .993 | .116 |
|  | Politeness | -.07 | .249 | -.19 | .05 |  |  |  |  |  |  | -.01 | .847 | -.13 | .11 |  |  |  |  |  |  | .988 | .123 |
|  | Compassion | -.12 | .175 | -.29 | .05 |  | -.13 | .085 | -.29 | .02 |  | -.04 | .603 | -.21 | .12 |  | -.05 | .586 | -.22 | .12 |  | .991 | .116 |
|  | Conscientiousness | -.06 | .488 | -.23 | .11 |  | -.12 | .118 | -.27 | .03 |  | .02 | .800 | -.15 | .19 |  | -.04 | .683 | -.21 | .14 |  | .990 | .117 |
|  | Industriousness | .001 | .987 | -.12 | .12 |  |  |  |  |  |  | .03 | .662 | -.10 | .15 |  |  |  |  |  |  | .985 | .130 |
|  | Orderliness | -.07 | .419 | -.24 | .10 |  | -.13 | .099 | -.28 | .02 |  | -.02 | .835 | -.19 | .15 |  | -.03 | .763 | -.20 | .15 |  | .986 | .114 |
|  | Neuroticism | -.06 | .557 | -.25 | .14 |  | -.09 | .321 | -.27 | .09 |  | -.09 | .323 | -.26 | .09 |  | **-.21** | **.027** | **-.40** | **-.02** |  | .974 | .149 |
|  | Withdrawal | -.06 | .511 | -.25 | .12 |  | -.08 | .379 | -.25 | .09 |  | **-.18** | **.035** | **-.35** | **-.01** |  | -.17 | .067 | -.36 | .01 |  | .975 | .145 |
|  | Volatility | -.04 | .649 | -.24 | .15 |  | -.12 | .177 | -.30 | .06 |  | .03 | .750 | -.14 | .20 |  | **-.20** | **.031** | **-.39** | **-.02** |  | .969 | .143 |
|  | Extraversion | -.06 | .338 | -.19 | .07 |  |  |  |  |  |  | .08 | .259 | -.06 | .21 |  |  |  |  |  |  | .990 | .125 |
|  | Assertiveness | -.10 | .234 | -.28 | .07 |  | -.08 | .311 | -.24 | .08 |  | .08 | .380 | -.09 | .25 |  | .04 | .690 | -.14 | .21 |  | .989 | .122 |
|  | Enthusiasm | -.08 | .248 | -.20 | .05 |  |  |  |  |  |  | .06 | .339 | -.07 | .19 |  |  |  |  |  |  | .989 | .118 |
| Only Meat Consumption | Openness/Intellect | **-.17** | **.002** | **-.28** | **-.06** |  |  |  |  |  |  | -.07 | .228 | -.18 | .04 |  |  |  |  |  |  | .991 | .123 |
|  | Openness | **-.16** | **.006** | **-.27** | **-.05** |  |  |  |  |  |  | -.02 | .769 | -.13 | .10 |  |  |  |  |  |  | .984 | .121 |
|  | Intellect | **-.13** | **.021** | **-.24** | **-.02** |  |  |  |  |  |  | -.09 | .130 | -.20 | .03 |  |  |  |  |  |  | .990 | .127 |
|  | Agreeableness | -.09 | .121 | -.20 | .02 |  |  |  |  |  |  | .03 | .588 | -.08 | .14 |  |  |  |  |  |  | .994 | .124 |
|  | Politeness | -.06 | .336 | -.17 | .06 |  |  |  |  |  |  | .05 | .360 | -.06 | .17 |  |  |  |  |  |  | .990 | .124 |
|  | Compassion | -.10 | .080 | -.21 | .01 |  |  |  |  |  |  | .004 | .939 | -.11 | .12 |  |  |  |  |  |  | .992 | .124 |
|  | Conscientiousness | -.04 | .446 | -.16 | .07 |  |  |  |  |  |  | .04 | .543 | -.08 | .15 |  |  |  |  |  |  | .989 | .131 |
|  | Industriousness | -.03 | .598 | -.15 | .08 |  |  |  |  |  |  | .01 | .872 | -.11 | .13 |  |  |  |  |  |  | .986 | .138 |
|  | Orderliness | -.04 | .539 | -.15 | .08 |  |  |  |  |  |  | .04 | .478 | -.07 | .15 |  |  |  |  |  |  | .987 | .122 |
|  | Neuroticism | .05 | .398 | -.07 | .17 |  |  |  |  |  |  | -.04 | .466 | -.16 | .07 |  |  |  |  |  |  | .979 | .139 |
|  | Withdrawal | -.02 | .784 | -.13 | .10 |  |  |  |  |  |  | -.07 | .205 | -.19 | .04 |  |  |  |  |  |  | .978 | .142 |
|  | Volatility | .11 | .068 | -.01 | .22 |  |  |  |  |  |  | .00 | .999 | -.12 | .12 |  |  |  |  |  |  | .976 | .134 |
|  | Extraversion | -.07 | .222 | -.18 | .04 |  |  |  |  |  |  | .08 | .160 | -.03 | .19 |  |  |  |  |  |  | .991 | .128 |
|  | Assertiveness | -.07 | .219 | -.19 | .04 |  |  |  |  |  |  | .06 | .292 | -.05 | .18 |  |  |  |  |  |  | .988 | .129 |
|  | Enthusiasm | -.05 | .391 | -.16 | .06 |  |  |  |  |  |  | .08 | .187 | -.04 | .19 |  |  |  |  |  |  | .989 | .123 |
| Only Fish Consumption | Openness/Intellect | -.08 | .159 | -.19 | .03 |  |  |  |  |  |  | .01 | .921 | -.11 | .12 |  |  |  |  |  |  | .992 | .110 |
|  | Openness | .01 | .831 | -.10 | .13 |  |  |  |  |  |  | -.01 | .872 | -.12 | .10 |  |  |  |  |  |  | .986 | .108 |
|  | Intellect | **-.14** | **.009** | **-.25** | **-.04** |  |  |  |  |  |  | -.003 | .954 | -.11 | .11 |  |  |  |  |  |  | .991 | .116 |
|  | Agreeableness | -.06 | .250 | -.17 | .05 |  |  |  |  |  |  | -.06 | .268 | -.17 | .05 |  |  |  |  |  |  | .995 | .112 |
|  | Politeness | -.02 | .750 | -.13 | .09 |  |  |  |  |  |  | -.04 | .449 | -.15 | .07 |  |  |  |  |  |  | .991 | .112 |
|  | Compassion | -.09 | .124 | -.20 | .02 |  |  |  |  |  |  | -.06 | .252 | -.18 | .05 |  |  |  |  |  |  | .993 | .112 |
|  | Conscientiousness | .07 | .224 | -.04 | .18 |  |  |  |  |  |  | .05 | .346 | -.06 | .17 |  |  |  |  |  |  | .990 | .121 |
|  | Industriousness | .03 | .555 | -.08 | .15 |  |  |  |  |  |  | .07 | .239 | -.05 | .18 |  |  |  |  |  |  | .988 | .128 |
|  | Orderliness | .09 | .127 | -.02 | .19 |  |  |  |  |  |  | .03 | .578 | -.08 | .14 |  |  |  |  |  |  | .988 | .111 |
|  | Neuroticism | .05 | .424 | -.07 | .16 |  |  |  |  |  |  | -.03 | .557 | -.15 | .08 |  |  |  |  |  |  | .981 | .130 |
|  | Withdrawal | -.02 | .745 | -.13 | .09 |  |  |  |  |  |  | .002 | .978 | -.11 | .12 |  |  |  |  |  |  | .980 | .133 |
|  | Volatility | .10 | .075 | -.01 | .21 |  |  |  |  |  |  | -.06 | .289 | -.17 | .05 |  |  |  |  |  |  | .978 | .123 |
|  | Extraversion | -.02 | .734 | -.13 | .09 |  |  |  |  |  |  | .03 | .551 | -.08 | .15 |  |  |  |  |  |  | .992 | .116 |
|  | Assertiveness | .03 | .591 | -.08 | .14 |  |  |  |  |  |  | .06 | .314 | -.05 | .17 |  |  |  |  |  |  | .990 | .117 |
|  | Enthusiasm | -.06 | .299 | -.17 | .05 |  |  |  |  |  |  | .001 | .989 | -.11 | .11 |  |  |  |  |  |  | .991 | .112 |

*Note.* Bolded rows indicate statistically significant effects (i.e., *p* < .05); Constrained models only have one actor and partner effect reported under the “*Actor Effect (Higher Meat Consumer)*”, and “*Partner Effect (Higher Meat Consumer)*” headings; 95% Confidence intervals are reported; GFI = Goodness of Fit Index; SRMR = Standardized Root Mean Squared; Each model includes age, education, income, and gender as covariates.

## Figure S1

*Openness/Intellect, Openness, Intellect, and Compassion by Meat Consumption Split by Partners Meat Consumption Levels.*


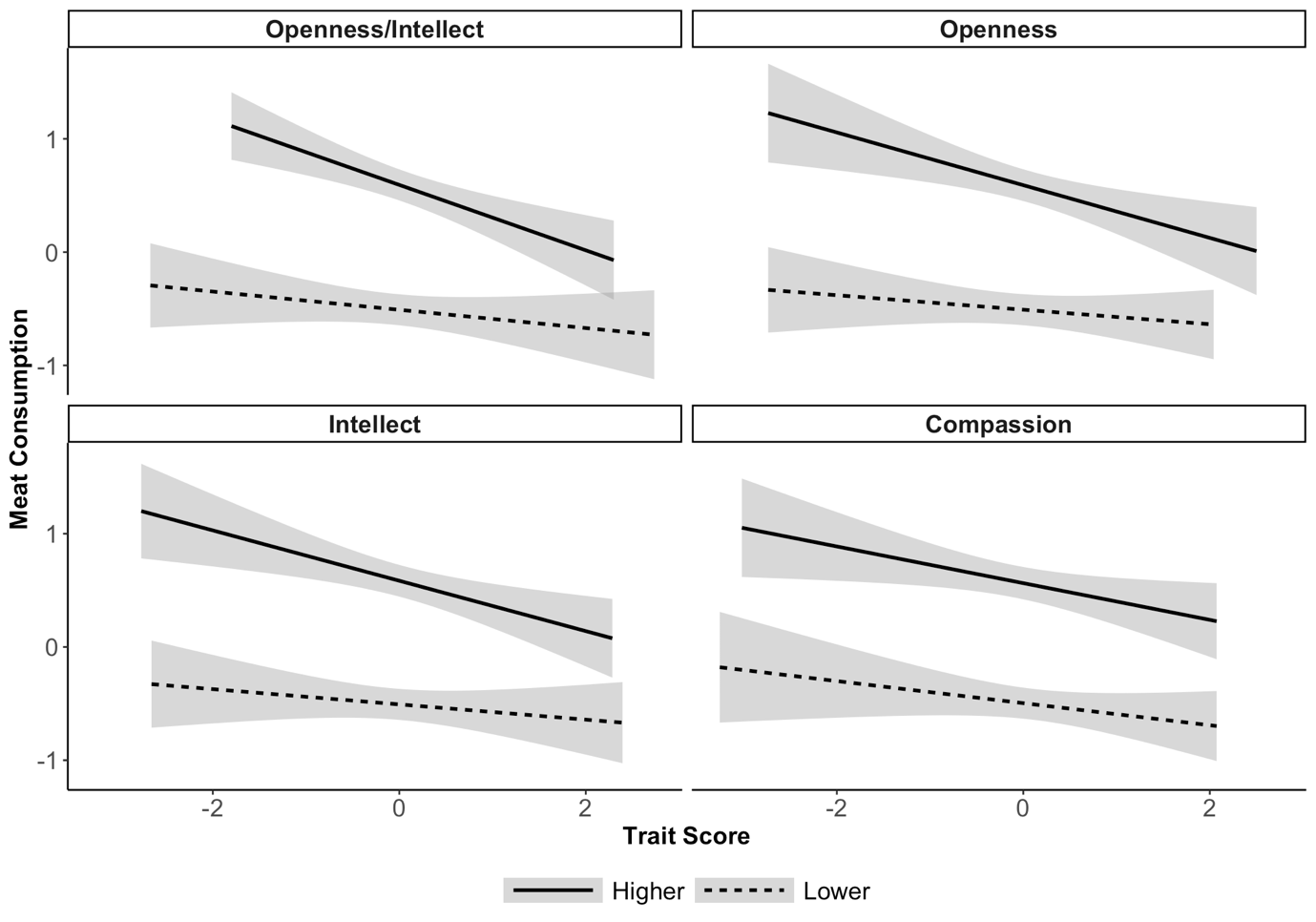


*Note.* Meat consumption and personality scores are standardized; Shaded areas represent the standard error of the mean; Personality scores are aggregated from self- and informant-reports; Meat consumption is aggregated from baseline and daily survey measures; Higher = Higher meat consuming partner; Lower = Lower meat consuming partner.
